# Damaged *Dickinsonia* specimens provide clues to Ediacaran vendobiont biology

**DOI:** 10.1371/journal.pone.0269638

**Published:** 2022-06-16

**Authors:** Gregory J. Retallack

**Affiliations:** Department of Earth Sciences, University of Oregon, Eugene, Oregon, United States of America; Birbal Sahni Institute of Palaeosciences, INDIA

## Abstract

Recently reported specimens of the enigmatic Ediacaran fossil *Dickinsonia* from Russia show damage and repair that provides evidence of how they grew, and of their biological affinities. Marginal and terminal areas of wilting deformation are necrotic zones separating regenerated growth, sometimes on two divergent axes, rather than a single axis. Necrotic zones of damage to *Dickinsonia* are not a thick scar or callus, like a wound or amputation. Nor are they smooth transitions to a regenerated tail or arm. The wilted necrotic zone is most like damage by freezing, salt, or sunburn of leaves and lichens, compatible with evidence of terrestrial habitat from associated frigid and gypsic paleosols. *Dickinsonia* did not regrow by postembryonic addition of modules from a subterminal or patterned growth zone as in earthworms, myriapods, trilobites, crustaceans, and lizards. Rather *Dickinsonia* postembryonic regrowth from sublethal damage was from microscopic apical and lateral meristems, as in plants and lichens. Considered as fungal, *Dickinsonia*, and perhaps others of Class Vendobionta, were more likely Glomeromycota or Mucoromycotina, rather than Ascomycota or Basidiomycota.

## Introduction

*Dickinsonia* is an iconic Ediacaran fossil best known from South Australia [1–3], but also from central Australia [4], around the Russian White Sea [5], Russian Urals [6], Ukraine [7], India [8], and China [9]. It is a problematic fossil with interpretations ranging from lichen [10], xenophyophore foraminifer [11], soft coral [12], sea jelly [13], annelid worm [14], placozoan [15], or extinct non-bilateran eumetazoan [2]. *Dickinsonia* has been assigned to the problematic group Vendobionta, variously considered a kingdom [16], phylum [17], or class [4]. Recent reports of “intravital damage” [5] now allow reassessment of biological affinities and growth of *Dickinsonia*. The principal hypothesis tested here is whether *Dickinsonia* grew by tissue patterning, like animals, or by meristems, like plants, and pseudomeristems, like fungi. Growth hypotheses based on living organisms of these three kingdoms are compared with observed damaged zones, and post-damage regenerated portions of *Dickinsonia*.

Specimens recovered from sublethal damage during life test these hypotheses because regeneration from injury is distinct in different kingdoms of organisms [18]. Plants regenerate from apical or lateral meristems to one side of damage callus [19, 20], and fungi have similar pseudomeristems [21, 22], but animals regenerate arms or tails from a blastema that does not leave a scar [23, 24]. Plants and fungi add modules from lateral meristems successively back from the apical meristem [19, 22], but animals add modules by cell patterning within subterminal growth zones [25–27]. Forms of damage are also distinct in the three kingdoms: swelling and scarring in animals [28–30], but browning, shrinkage, or wilting in plants [31–34] and fungi [21, 35, 36]. Wounded and regenerated Ediacaran fossils recently reported [5] can potentially reveal both biological affinities and mechanisms of growth of *Dickinsonia*.

## Materials and methods

**Fig 1:**
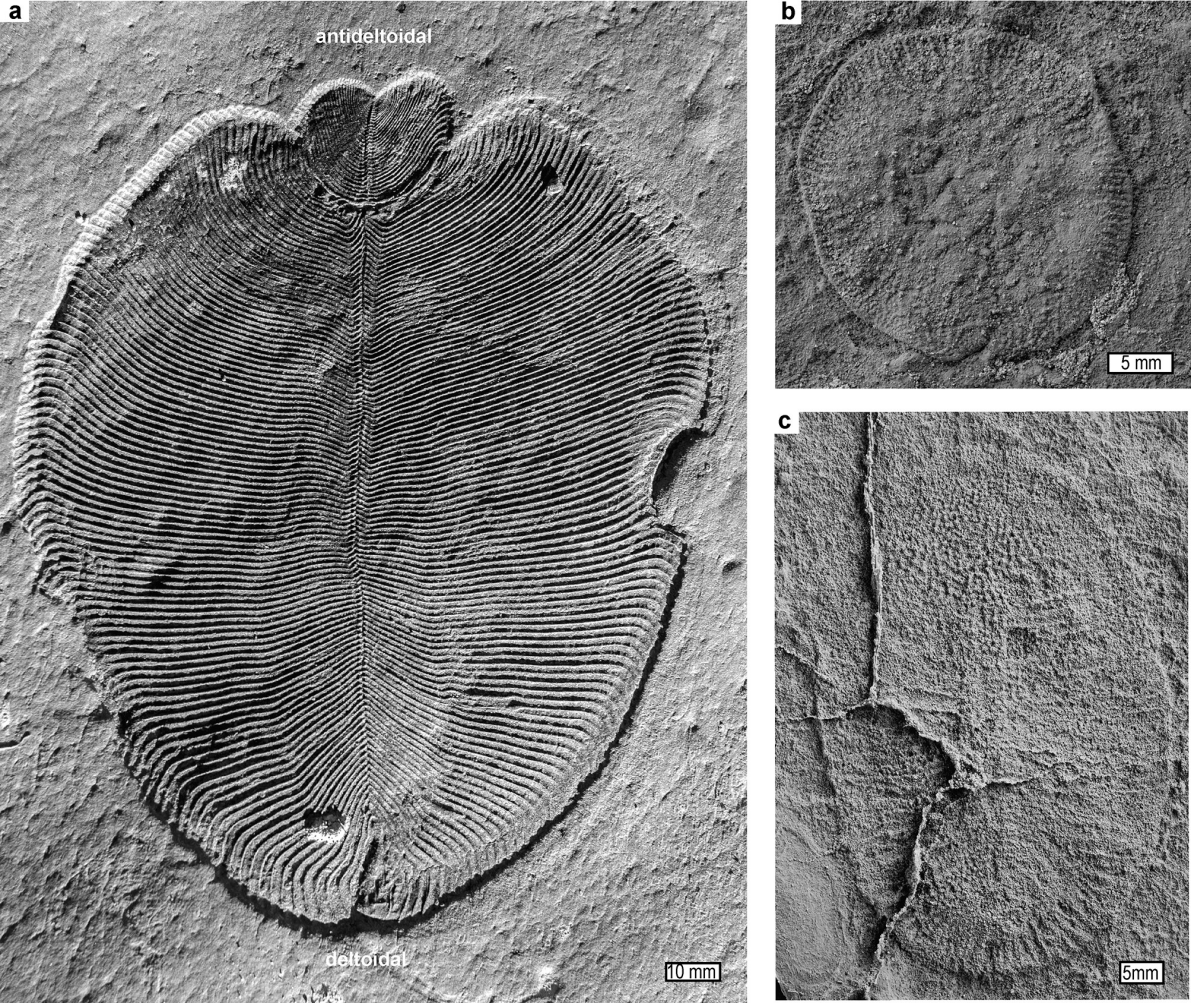
Fossil vendobionts from the Lyamtsa locality of Ediacaran, Ust Pinega Formation of the White Sea region: a, vendobiont *Dickinsonia menneri*; b, vendobiont *Yorgia waggoneri*; c, vendobiont *Dickinsonia tenuis*. Specimen numbers in the Paleontological Institute Moscow are PIN4716/5170 (a), PIN3993/5501 (b), PIN3993/850 (c), and images are courtesy of A. Ivantsov.

**Fig 2:**
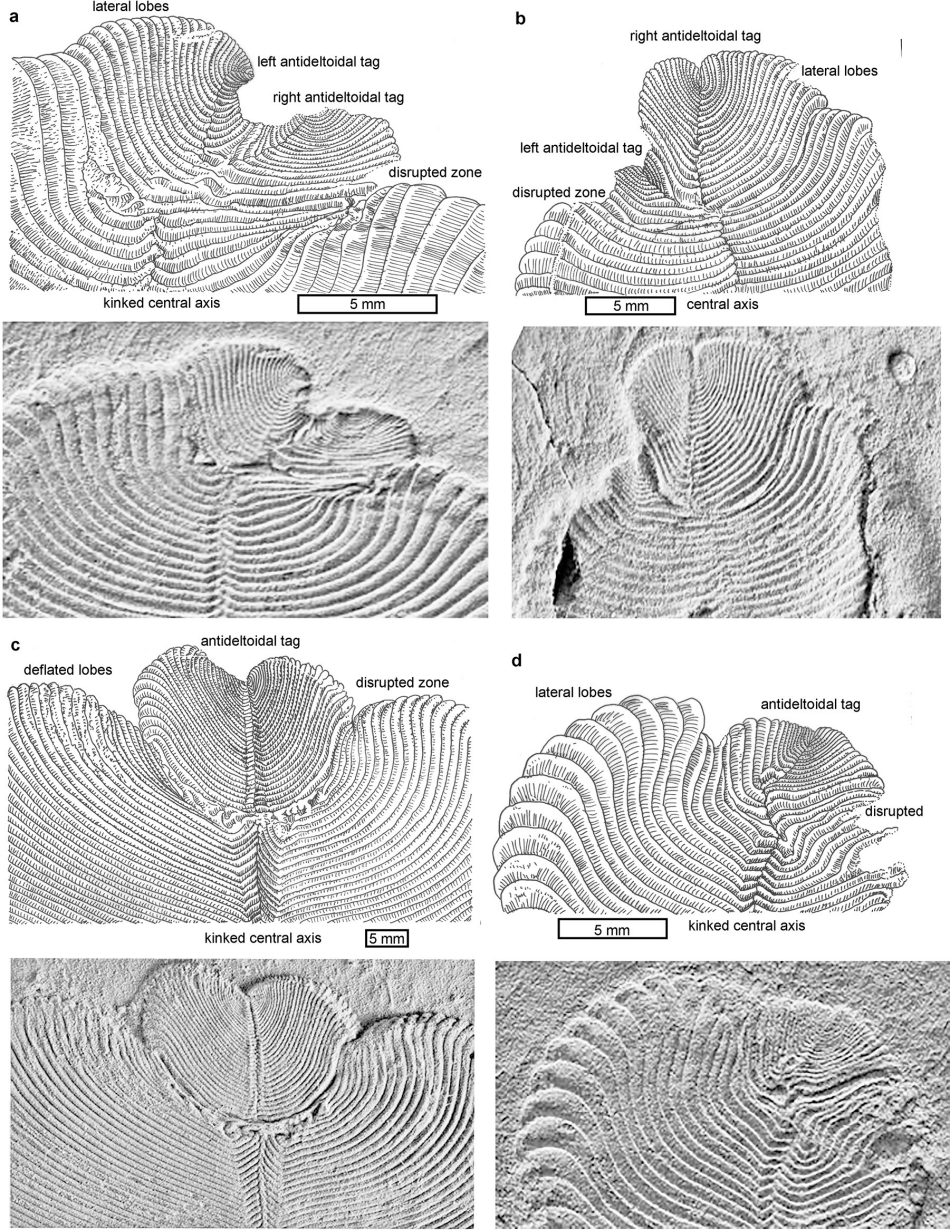
Recovery from damage to the antideltoidal end of *Dickinsonia menneri* from the Ediacaran, Ust Pinega Formation, at the Lyamtsa locality of the southeastern White Sea region Russia, using non-genetic terminology [40]. **Fig 2C** is the same specimen as **Fig 1A**. Specimens are PIN4176/5188 (a), PIN4176/5146 (b), PIN4176/5170 (c), PIN4176/5182 (d).

*Dickinsonia menneri* and *D*. *tenuis* fossils discussed here (Figs 1 and 2) are from the Ediacaran, Ust Pinega Formation at the Lyamtsa locality of the southeastern White Sea region of Russia, and are all reposited in the Paleontological Institute of Moscow [37, 38]. Of particular interest are specimens with unusual morphology interpreted as “intravital damage”, or non-fatal wounding that was later repaired [5, 39]. This paper is a redescription of the damage within the context of a non-genetic polarity terminology specific to *Dickinsonia* [40], based on excellent photographs and sketches provided by Andrey Ivantsov. It is a wide-ranging search among living organisms for anything morphologically comparable with the disrupted zone, and regrown addition. Implications of these comparisons are then considered within the context of other evidence on the biology and paleoenvironments of *Dickinsonia*.

## Observations of damaged and recovered *Dickinsonia*

Hoekzema et al. [40] propose useful non-genetic terms for the distinctly different ends of *Dickinsonia*: deltoidal region for the end with a triangular flat region like the keystone of an arch, and antideltoidal region for the other end of invaginated modules (Fig 1A). This study is concerned with the antideltoidal region of specimens with extensive disrupted modules right across the fossil (Fig 2), especially “two-sided deformation” [5]. The disrupted zone is a highly deformed and wrinkled area between the main part of the fossil and an additional cordate or bilobed addition, here given the non-genetic name “antideltoidal tag”.

Transverse divisions of *Dickinsonia* have long been considered “segments” like those of annelids [14], but they rarely continue across the midline [1, 2, 41], where they are usually offset in zigzag fashion [16]. The term “module” suggested by Evans et al. [2] is preferred here, including mainly lateral modules. Whether deltoidal or antideltoidal modules can be considered basal or terminal modules, heads or holdfasts, is the central controversy addressed in this paper.

The interpretation of the disrupted zone by Ivantsov et al. [5] as “intravital damage” is accepted here as an assumption of this study, based on the continuation of the antideltoidal tag, or pair of tags. These specimens appear to be exceptional damage rather than regular or common growth interruptions, because very few specimens are known. Other specimens of *Dickinsonia* do not show recovery or repair of wrinkled or torn margins, but rather shredding to angular pieces, disruption by cracks extending into underlying sediment, stretching by sediment deformation, partial consumption by burrows or trails, excision of arcuate sections, or serial “footprints” from intermittent motion or transport [1–3, 8, 9, 42–45]. *Dickinsonia* did not necessarily move of its own accord, because the “footprints” may be “glacier mice”, or polsters frozen and driven by wind on melting ice [46]. These other fragments and deformed specimens reveal much about the tough integument and death of *Dickinsonia*, particularly a range of ductile to brittle behavior, interpreted here as degrees of freezing or desiccation of a normally pliable integument before burial.

The damaged Russian specimens are negative hyporeliefs on the soles of overlying slabs, as usual for *Dickinsonia*, and the disrupted wrinkled zone bulges to levels that would have been below the original upper surface. The bulges were depressions with flanking narrow ridges on the original body below the covering slab, and formed a zone of deformed shrinkage separating the antideltoidal tag, or tags. The bulges are wrinkled with high relief as if shrunken and desiccated, so differ fundamentally from Ediacaran non-resistant or sunken compressions of Wade [47], best known from *Nemiana* [48]. Burial compaction of *Nemiana* with jellylike consistence resulted in a convex hyporelief on the overlying slab, but *Dickinsonia* was far from jellylike as revealed by specimens lacerated into brittle shards [3, 45]. *Dickinsonia* fossils are concave hyporeliefs generally taken as evidence of a compaction-resistant biopolymer [47, 48]. The distinction between levels of the disrupted zone and the rest of *Dickinsonia* may reflect loss of compaction-resistance by pre-burial wilting, shrinkage, or hollowing out within that zone [42].

In some cases, there is a single antideltoidal tag (Fig 2C and 2D), but in other cases there are two tags (Fig 2A and 2B). Paired tags diverge laterally, then curve parallel with the original axis toward the end. One of the antideltoidal tags originates laterally one module before the other tag, just as modules on the main body alternate along the midline.

Antideltoidal tags separated by a disrupted zone are the main puzzle addressed by this paper, but also notable is a kinked central axis in three of the four specimens illustrated in Fig 2, as if lesser damage preceded the more extensively disrupted zone. Similar shrinkage and buckling is also seen in other marginal areas of this Russian collection of *Dickinsonia* [5]. Another anomalous feature in these Russian *Dickinsonia* (Fig 1C) and the similar genus *Yorgia* (Fig 1B) is a pustulose texture of spherical bodies within an upper integument [38].

## Interpretation of *Dickinsonia* disrupted zone

### Infection

Infection of animals and plants by pathogens and parasites usually includes swelling into blisters or galls preserved in fossil leaves, shells and bones [49]. These swellings in soft-bodied organisms are thick callus or scar tissue [28, 29, 50]. The pustulose texture of the upper surface of some *Dickinsonia* (Fig 1B and 1C) may be infection comparable with tar spot fungus, *Rhytisma acerinum*; [51]. However other explanations are also plausible, for example, as a tubercular ornament [39], or as reproductive structures [10]. Infection is not a good explanation for the observed withered and shrunken, disrupted zone of *Dickinsonia*, because the disrupted zone is neither pustulose, swollen, nor hollow (Figs 1 and 2). Furthermore, the whole organism would not have been infected, because isomers before and after the disruption are unaffected.

### Atavism

Atavisms are genetic mistakes that recapitulate evolutionary history, such as tails in humans [52], extra digits in horse feet [53], or multiheaded cnidarian polyps [54]. Could antideltoidal tags in late Ediacaran *Dickinsonia* be rare outgrowths of lobes recapitulating multilobed middle Ediacaran vendobionts? No atavisms have previously been noted in Ediacaran fossils, but a plausible case is *Hylaecullulus fordi*, which has a complex branching system of fronds? Unlike the *Dickinsonia* specimens discussed here (Figs 1 and 2), adventitious fronds of *Hylaecullulus*, are not separated by a disrupted zone, and are part of a coherent fractal branch system [55]. The relationship of *Hylaecullulus* and other rangeomorphs to *Dickinsonia* is uncertain [56]. Disrupted zones of weakness separating supernumary elements are not seen in growths that could be considered atavisms in modern or ancient examples [52–54]. The post-damage antideltoidal tags of *Dickinsonia* have disrupted zones distinct from atavisms.

### Laceration scar or callus

Laceration of animals creates scars [57], and in plants it creates callus or resin [32, 49]. Injury to hard tissue such as teeth or shells also produces swelling and deformation of symmetry [29, 49]. Predation damage is unlikely for *Dickinsonia* given variable expression of deformation and lack of known large predators in the Ediacaran [5]. Comparable deformation is lacking in *Dickinsonia* consumed along worm trails [43]. In sponges, severe dismemberment to small fragments is repaired without scars or deformed zones [58, 59]. Scarless repair of injury is also found in placozoans, planarian worms, comb jellies, and cnidarian polyps [18, 60, 61], again unlike *Dickinsonia*. Scarless whole-body regeneration is not found in vertebrates [62], and scarless limb regeneration is lost in frogs after metamorphosis [24]. Recovery by scar and callus formation is mainly found in large perennial organisms [32, 49, 57], and *Dickinsonia* was both large and perennial compared with associated fossils [63]. Scar and callus tissues form compact protruding seals, unlike the withered, disrupted zone of *Dickinsonia*, or the clean edges of dismembered *Dickinsonia* [2, 44, 45].

### Frost, sunburn or salt injury

**Fig 3:**
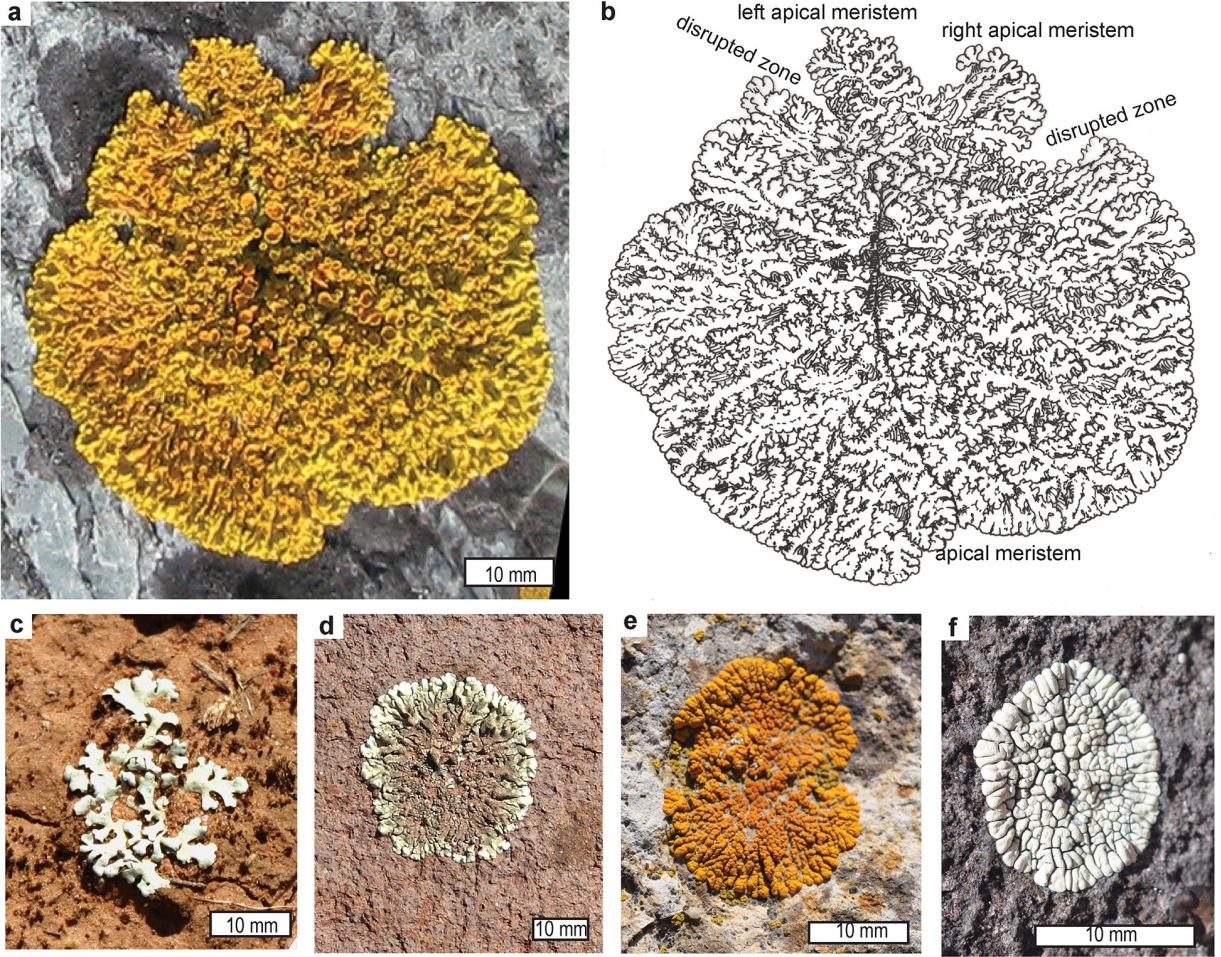
Modern lichens showing apical and lateral meristematic mode of growth: *Caloplaca verruculifera* image (a), and interpretive sketch of lobe disposition with apothecia removed (b), from rock platform exposure of Ediacaran, Gaskiers Formation, 4 m above sea level, St Marys, Newfoundland; lichen *Xanthoparmelia terrestris* on red soil between belah (*Casuarina cristata*) trees at Back Creek State Forest 16 km east of West Wyalong, N.S.W., Australia (c), *Xanthoparmelia plittsi* (d) and *Polycauliona ignea* (e) on welded tuff of Oligocene, John Day Formation on Carroll Rim, Painted Hills State Park, Oregon, and *Dimelaena oreina* (f) on Steens Basalt at Paisley Caves, Oregon.

These three distinctly different causes of injury create similar effects in fungi and plants, distinct from their effects in animals. In humans, frostbite produces swelling, and then death of tissue, best treated by amputation or scraping back to live tissue, that then is a scar [28]. Damage of humans by salt and sunburn also causes swelling, blisters, peeling skin, and can result in scars [64], which also are unlike the disrupted zone of *Dickinsonia*. Freezing, hypersalinity, and sunburn do not create local disrupted growth zones in aquatic creatures, such as sea jellies or polychaetes, but kill, desiccate, and wither the whole organism [65, 66]. Frost, sunburn and salt injury of lichens results in death of the photosynthetic layer on thallus margins, and shrinking and death of the growth apex down to the hypothallus [35, 36]. The apex is then replaced by one or more lateral meristems to form an apical tag or tags elaborated from apical threads beyond the wilted and necrotic zone (Fig 3A and 3B), broadly similar to those observed in *Dickinsonia* (Fig 2). There has been controversy for *Dickinsonia* in interpreting a rim around the fossils as a hypothallus with branching hyphae [42], as scrape impressions in the sediment of the margin of a shrunken individual [14], as signs of self-propelled incipient motion [45], or as incipient dislodgement by basal freezing [46]. In plant leaves, frost, sunburn and salt injury shrink both palisade and mesenchyme cells of the margin. With loss of chloroplasts and chromophores, this results in browning, thinning, curling, and wrinkling [31–34, 67, 68].

Of the four alternatives considered here, sunburned and salt damaged leaves and lichens are the best modern analogy for the terminal disrupted zone of *Dickinsonia*. Unlike animal repair, there was only minor deformation anticipating the break-line, rather than distributed deformation, axial more than peripheral addition, and failure to completely restore the overall shape. By this analogy, antideltoidal ends of *Dickinsonia* were growth zones, and deltoidal ends were holdfasts or growth initials.

## Interpretation of *Dickinsonia* antideltoidal tag

### Budding

A common form of asexual reproduction in animals is budding, well known in living placozoans [69], sponges [70, 71], and cnidarians [72]. Budding also is preserved in fossil invertebrates [49, 73, 74]. Budding starts as an outgrowth from a stolon or other narrow part of the parent, then grows into another undeformed individual attached by an undisrupted narrow stalk. The newly budded individual is a replica of the parent, not a continuation of modified modules of the adult, as in the antideltoidal tags described here (Fig 2). Stolons are often long, but even short stolons have a constriction that allows the bud to detach from the parent, unlike the antideltoidal tag nestled within the end of *Dickinsonia* (Fig 2).

### Limb regeneration

Limb and tail regeneration is well known in starfish [23] and amphibians [24], but regeneration also is known from 18 additional animal phyla [62]. Up to six tails can be regenerated by lizards, in an unusual branching structure [75]. Scar-less regenerated limbs are also recorded among fossil lizards and decapods, and are especially obvious when still smaller than the original limbs and tails [49]. Sponges regenerate entire colonies from small pieces with no evident scarring or damaged zones [58, 59], and so can placozoans, planarian worms, comb jellies, cnidarian polyps and molluscs [18, 60–62, 76]. Scar-free regeneration of limbs is achieved through many processes, including immune system removal of damage, cell dedifferentiation, cell transdifferentiation, and cell patterning in a broad blastema, rather than from a narrow meristem [18, 24]. Lack of a disrupted zone in regenerated animal parts is distinct from the antideltoidal tag defined by a disrupted zone in *Dickinsonia* (Figs 1 and 2).

### Subterminal growth zone

**Fig 4:**
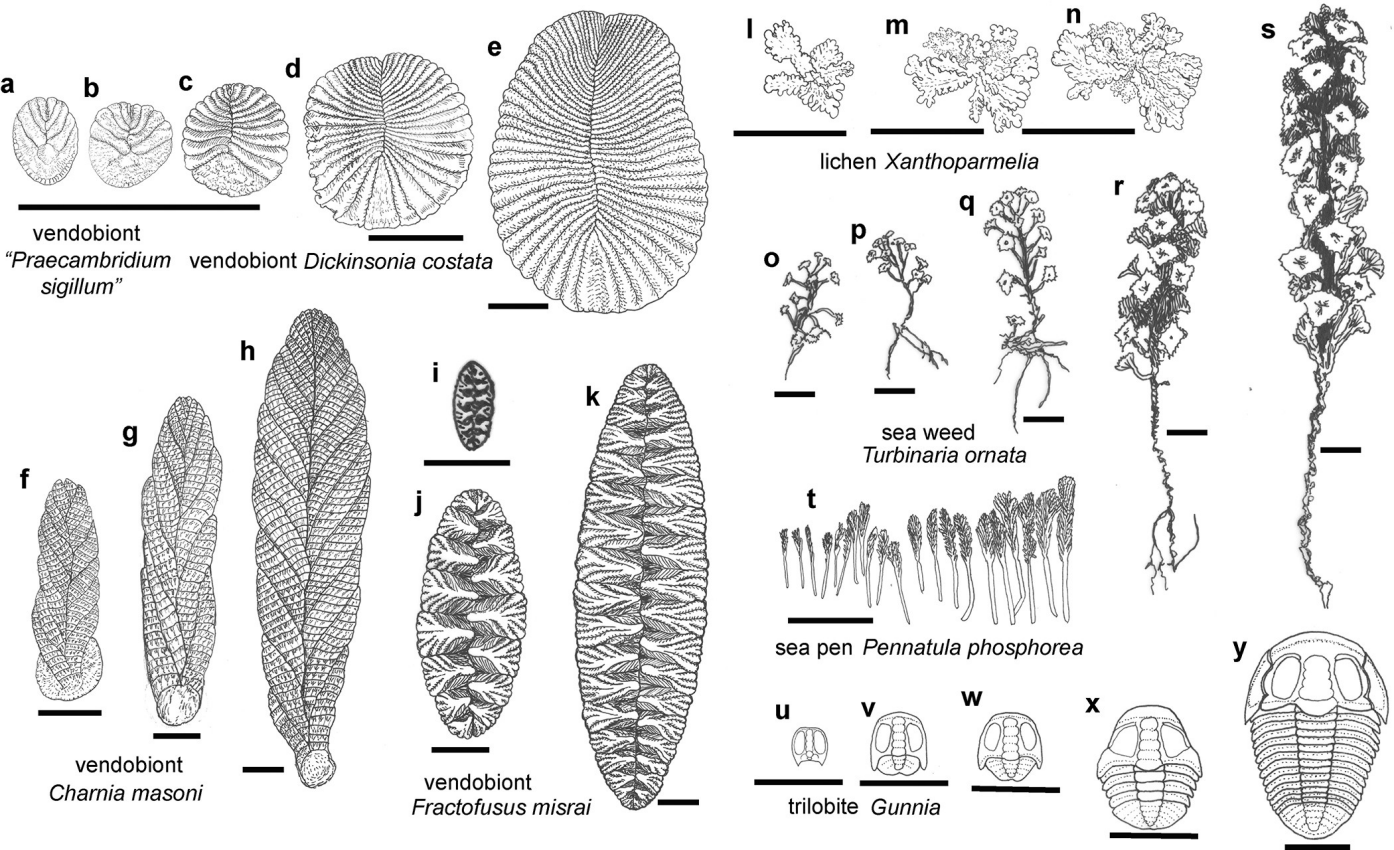
Growth series of vendobionts (a-k), living organisms (i-t), and trilobite (u-y): a-b, “*Praecambridium sigillum”* from the Ediacara Member of the Rawnsley Quartzite Ediacara Hills, South Australia [94]: c-e, *Dickinsonia costata* from the Ediacara Member of the Rawnsley Quartzite Ediacara Hills, South Australia [14, 96]; f-h, *Charnia masoni* [55, 86] from the Drook Formation at Drook Newfoundland (f), Mistaken Point Formation at Mistaken Point, Newfoundland (g), and Bradgate Formation in Charnwood Forest, England (h): i-k, *Fractofusus misrai* from the Mistaken Point Formation at Mistaken Point, Newfoundland [114, 146]: l-n, crustose lichen *Xanthoparmelia* sp. indet, on a granite tombstone, in successive years 8 Nov. 2005, 27 Sept 2006 and 21 May 2007, Petersham, Massachusetts [81]; o-s, phaeophyte alga *Turbinaria ornata* from Moorea, French Polynesia [88]; t, sea pen *Pennatula phosphorea* from the North Sea, UK [26]; u-y, trilobite *Gunnia* sp. indet. from the Middle Cambrian, Gaotai Formation, from Balang, Guizhou Province, China [27]. Scale bars all 10 mm.

**Fig 5:**
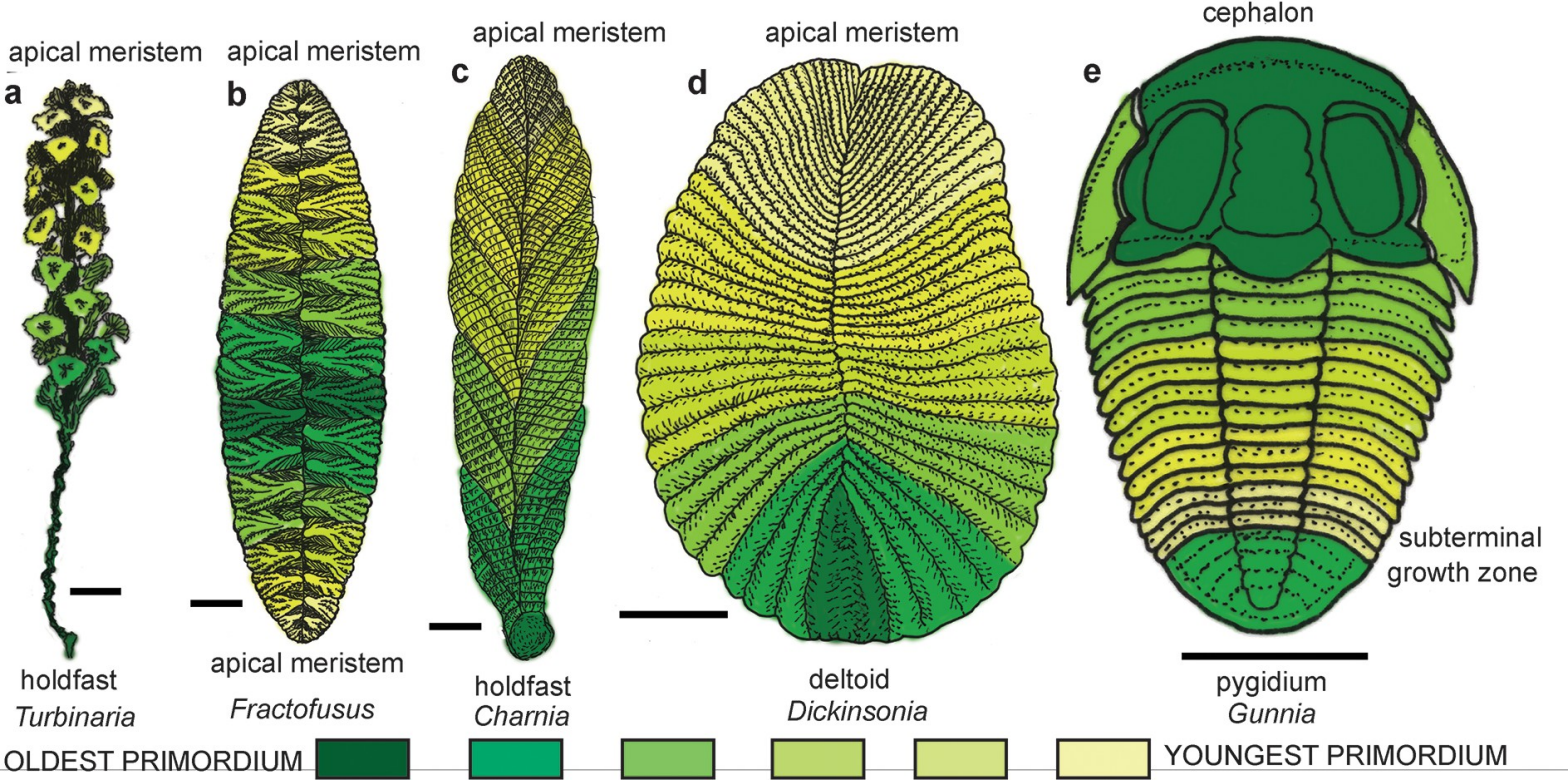
Interpreted relative age of mature examples of vendobionts, sea weeds, sea pens and trilobite based on Fig 3: a, phaeophyte alga *Turbinaria ornata* from Moorea, French Polynesia [88]; b, vendobiont *Fractofusus misrai* from the Mistaken Point Formation at Mistaken Point, Newfoundland [114]; c, vendobiont *Charnia masoni* from Bradgate Formation at Charnwood Forest, England [86]; d, vendobiont *Dickinsonia costata* from the Ediacara Member of the Rawnsley Quartzite Ediacara Hills, South Australia [14]; e, trilobite *Gunnia* sp. indet. from the Middle Cambrian, Gaotai Formation, from Balang, Guizhou Province, China [27]. Scale bars all 10 mm.

In animals, body parts are specified by cell patterning in the developing embryo, including terminal growth of tails [41, 77]. Postembryonic terminal regeneration of vertebrate tails is also achieved by cell patterning in cartilage of the elongating blastema cone, rather than terminal or intercalary addition of ossified vertebrae [78]. An animal model of interstitial regeneration of *Dickinsonia* advocated by Ivantsov et al. [5] would have resulted in seamless tail regrowth [75], unlike the fossils discussed here. Segments also are added during postembryonic growth from a subterminal growth zone in sea pens [26], trilobites [27], millipedes [79], and earthworms [25]. The growth zone is subterminal in animals, because the terminal segment is established by embryonic cell patterning count-down early after the head. That terminal segment is variously known as pygidium in trilobites [27], and periproct in millipedes [79], and earthworms [25]. The pygidium and periproct are at least millimetric in size, and would have been preserved in *Dickinsonia* with fine-grained clayey matrix like the Russian specimens. They have never been found, and the antideltoidal meristem was evidently microscopic. The antideltoidal tags in *Dickinsonia* are at the end and separated from the rest of the body by the disrupted zone, unlike subterminal growth zones in animals (Figs 4 and 5).

### Apical and lateral meristems

A system of apical and lateral pseudomeristems is found in fungi [21, 22, 80, 81] and a system of meristems in plants [19, 20]. Pseudomeristems and pseudoparenchyma of lichens mature to appear very similar to meristems and parenchyma, but form by septation of hyphae rather than proliferation of cubic cells [82]. The apical meristem is the terminus of the main shoot, but lateral meristems are the tips of branches. These laterals may emerge as leaders when the apical meristem is lethally damaged [19, 83]. Growth on two opposed apical pseudomeristems and numerous radial laterals explains the growth of lichens (Fig 3), and the Ediacaran fossil *Fractofusus* (Fig 4), and such opposed meristems are known in non-vascular plants [84, 85]. The growth pattern of other Ediacaran fossils, such as *Charnia* [26, 86, 87], show a holdfast at the base, and an apical meristem at the other end (Fig 4), most like algae such as *Turbinaria* [88], Growth from the anti-holdfast end is also noted by Dunn et al. [89], who also propose, without justification, continuing growth in the stalk. By the contrasting rangeomorph model of Antcliffe and Brasier [26], the deltoidal region of *Dickinsonia* is a holdfast rather than a head, and the antideltoidal region is an apical meristem or pseudomeristem. The deltoid holdfast interpretation is especially suggested by the rounded terminal module of “*Praecambridium sigillum*” (Fig 4), proposed as juveniles of *Dickinsonia* by Runnegar [14]. Twin antideltoidal tags can thus be explained as axial lateral meristems emerging after damage of the apical meristem. The two antideltoidals alternate like all the lateral modules of the zigzag central suture representing alternate fractal growth. Comparable leaders are created by removal of the terminal meristem during pollarding of trees [83].

## Growth of *Dickinsonia*

*Dickinsonia* grew with addition of modules (Fig 4), but different growth alternatives have been proposed. Is the deltoidal end a head or a holdfast? Is the deltoidal end anterior or posterior? Hoekzema et al. [40] plotted both antideltoidal-first and deltoidal-first growth models and found changing rates of module length through life to maintain an ovoid overall shape. The deltoidal-first pattern is a less extreme change in relative module length, so they interpreted the deltoidal as the oldest part, thus literally anterior, and the antideltoidal part as the youngest part, thus literally posterior. Another meaning of anterior is the direction of movement, such as head-first in vertebrates, but direction of movement is unclear in serial imprints of *Dickinsonia* [2], misinterpreted as trails [46, 90]. Hoekzema et al. [40] rejected the antideltoidal-first hypothesis because that “trend in our studied specimens is not unidirectional (as would be expected in an organism with a well-regulated growth programme).” Hoekzema et al. [40] also assumed that it was an animal which grew by subterminal addition, so that the deltoidal would be the terminal posterior module comparable with a periproct or pygidium of trilobites (Fig 4). Dunn et al. [89] mark both the deltoidal and antideltoidal as the oldest parts (both anterior? or unresolved?), with modules interpolated between. Dunn et al. [89] also align the antideltoidal with an insect head (anterior) and deltoidal with an insect tail (posterior). By both interpretations, *Dickinsonia* was an animal up to 1.4 m long [42] with a microscopic head: a head too small to be observed in any known fossil impression. Growth from the deltoidal end, or subterminal to it, is falsified by antideltoidal but not deltoidal regrowth, represented by antideltoidal tags [5, 45].

Another view of Runnegar [14], followed by others [5, 41, 91–93], regards the deltoidal as an anterior “head”, and the antideltoidal as a posterior “tail” most like those of annelids and insects, rather than postanal tails of lizards and other vertebrates. Runnegar [14] supported this interpretation by adding “*Praecambridium sigillum*” [94], with its disc on one end, as a juvenile to a growth series of *Dickinsonia* (Fig 3). Others [95] have also argued that *Dickinsonia* fossils with proportionally widest deltoids were youngest. This interpretation is a holdover from an earlier view of *Dickinsonia* as an annelid, including interpretation of the midline as a gut connecting a deltoidal mouth and antideltoidal anus [14, 96]. My own examination of hundreds of specimens has been unable to identify digestive anatomy [42], and there is widespread agreement that *Dickinsonia* lacked digestive tract, mouth, anus, or periproct [15, 44, 93]. Elongation of the antideltoidal end in progressively older specimens is supported by both growth series [14, 42] and antideltoidal tags [5, 45]. Nor do series of impressions reveal that *Dickinsonia* moved of its own accord in the direction of the deltoidal region [2], because that older region would have been more heavily frozen and driven by wind after basal melting [46].

A third view of Retallack [10, 42, 48] regards the deltoidal as an anterior holdfast, supporting an antideltoidal posterior axis, as in Ediacaran fronds such as *Charnia* (Figs 4F–4H). The deltoid may have originally been circular and the full width of the body (14), but the deltoid diminished in relative width with addition of terminal modules (Figs 4A–4E). This deltoid-holdfast interpretation explains antideltoidal tags and disrupted zones as sublethal interruption of terminal growth (Fig 2). Antideltoidal regeneration can be explained as due to a system of apical and lateral meristems as documented in lichens [21, 22, 81] and plants [19, 20, 97]. Paired antideltoidal tags may be lateral meristems resuming growth after damage of posterior modules and death of the terminal meristem. *Dickinsonia* rarely shows true segmentation of creases right across the body [41, 93], but commonly had a glide symmetry of modules alternating along a midline [16, 98], including divergent paired antideltoidal tags (Fig 2A and 2B). Wade [96] argued for a small antideltoidal terminal module of *Dickinsonia* like the periproct of polychaetes, which she considered modern descendants of *Dickinsonia*. No such terminal module has been demonstrated [40, 89]. The generative point of the antideltoidal end was microscopic and flanked by small, thin, modules, like an apical meristem flanked by young, developing, podetia or leaves of fungi and plants [19, 22].

**Fig 6:**
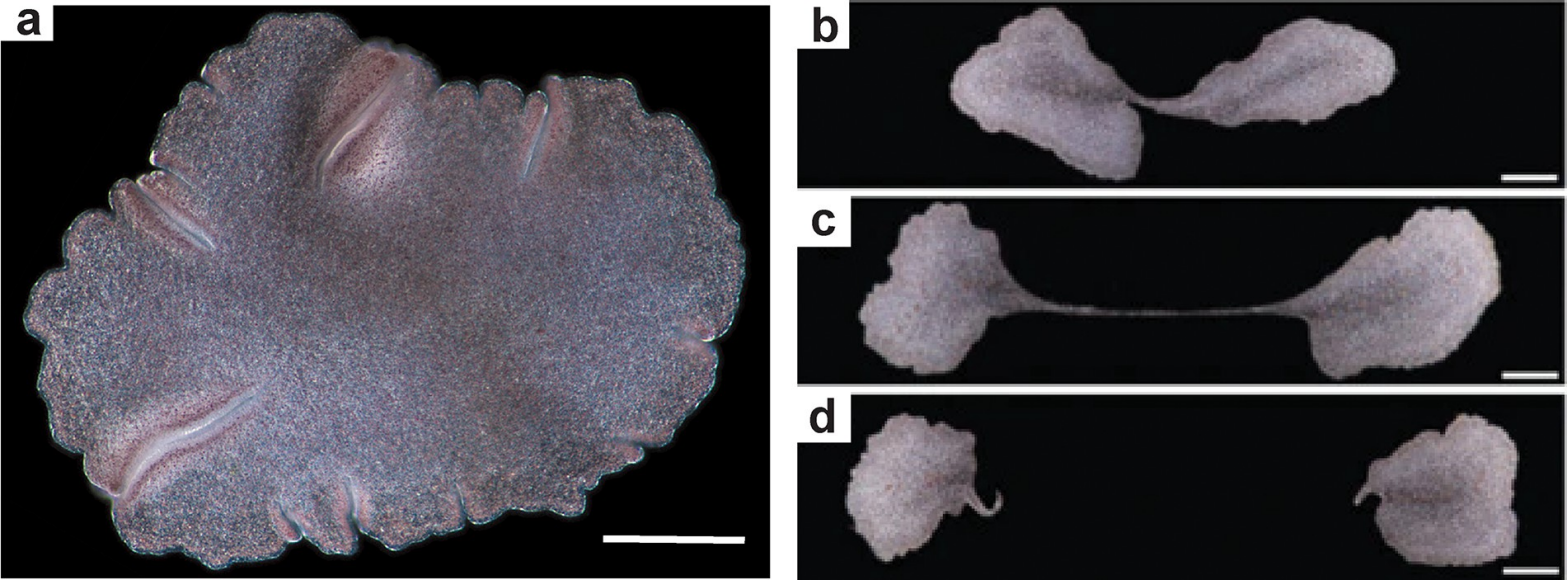
The placozoan *Trichoplax adhaerens* (a), and its reproduction by fission (b-d). Scales in panels a-d are 200 μm. From [60] with permission.

The developmental implications of suggested placozoan affinities for *Dickinsonia* [15] are unclear, because living placozoans such as *Trichoplax* lack segmentation and anterior-posterior differentiation (Fig 6A). *Trichoplax* does have dorso-ventral differentiation, only 4 cell types, and a single HOX gene [60, 99]. *Trichoplax* alternates between spherical and flattened bodies formed by radial cell-division, and can divide into two halves separated by a thread or stolon [60, 69, 100], which is eventually severed (Fig 6B–6D). This unique growth form may be relevant to a placozoan interpretation of *Dickinsonia* if placozoans represent an evolutionary transition from fungi to metazoans [99, 100], because then apical and lateral meristematic growth of fungi and plants would have been lost before evolution of subterminal addition in animals [25, 27, 79]. However, the idea of placozoans as the earliest diverging animal lineage is now doubtful, with animal derivation from unicellular choanoflagellates more likely [101, 102].

More cogent evidence for the distinctly different development of plants, fungi, and animals is phylogenomic. The topology of molecular trees has varied greatly over the years, but many agree that plants, fungi, and animals developed multicellularity independently from unicellular ancestors [103–105]. The three kingdoms also have different genes for development: KNOX and MADS for plant meristematic growth, MADS for fungal and lichen pseudomeristematic growth, and HOX for animal cell patterning [99, 106–108]. A less compelling generalization, because of exceptions such as metamorphosis and long-lived animals, is that animal development is mainly embryonic, and plant-fungus development is mainly postembryonic [109]. This generalization is reflected in the generalization of determinate growth for animals, but indeterminate growth for plants and fungi. Elephants, alligators and sea turtles have been considered an exception to this generalization, but comprehensive studies have demonstrated that all three are determinate, with a distinct age of no further growth [110–112]. Nevertheless, animals such as placozoans and planarian worms may have indeterminate growth [113]. Indeterminate growth has been demonstrated for *Dickinsonia* [42].

The scheme of development for *Dickinsoni*a preferred here is outlined in Fig 5, in which darkest hues are oldest and anterior, and the lightest hues are youngest and posterior in terms of the branch order of their module primordia. Each module has a lateral meristem which is likely a diffuse marginal meristem like that of developing leaf, rather than a separate shoot [83]. In a meristematic system, Ediacaran *Fractofusus* (Fig 5B) [114] had divergent apical meristems like the development of lichens (Fig 3), but Ediacaran *Charnia* (Fig 5C) [26] had a single apical meristem, like brown algae (Fig 5A) [88], and lichens such as *Cladonia* [22]. So the question is whether *Dickinsonia* added modules from a terminal meristem like an alga or fungus, or in a subterminal growth zone like a trilobite (Fig 5E)? Antideltoidal tags and multiple regenerative axes are evidence that *Dickinsonia* had a meristematic system like a plant or fungus. Preservation of a disrupted zone between normally formed parts of large specimens [5] also implies temporary interruption of indeterminate growth of a perennial structure, rather than injury of a short-lived creature with limited determinate growth. *Dickinsonia* did not grow and regenerate like arthropods or annelids, nor like sponges or placozoans.

## Other evidence for biology of *Dickinsonia*

### Sedimentary context

*Dickinsonia* in South Australia has been interpreted as a shallow marine or intertidal creature, thrown up by storms onto the shore [115, 116], but revised facies analysis interpreted them as entirely submarine [117]. Comparable facies analysis of Russian *Dickinsonia* found them in middle to upper shoreface prodelta facies [118]. Doubts about marine habitats came from the discovery in South Australia of *Dickinsonia* atop paleosols, showing soil textures, carbonate nodules with pedogenic stable isotopic covariance, desert rose pseudomorphs, periglacial convolutions, and hydrolytic chemical weathering profiles [48.63, 119]. Paleosols directly below *Dickinsonia* have also been found in central Australia [120], India [6], and Russia [8, 90]. Drab-haloed threads down into red paleosols below *Dickinsonia* [6, 63] are *Prasinema*: traces of mycelia or rope-forming cyanobacteria common in paleosols [121–123]. These subvertical drab threads disturb bedding and create massive red beds in the field and in thin sections [48], unlike the laminated microbial mat interpretation of the same beds [2, 124]. Ediacaran “Mattressland” vendobionts of red beds [120] may be contrasted with Ediacaran grey stromatolitic carbonates and shaley turbidites with marine tubular fossils such as *Gaojianshania*, *Conotubus*, *Cloudina*, and *Namacalathus* of Ediacaran “Wormworld” [125–127].

Ediacaran paleosols include periglacial convolutions and ground ice as evidence for freezing [48, 90, 128], here proposed as a plausible explanation for disrupted zones of *Dickinsonia*. Gypsum desert roses in the paleosols [63, 116, 119] support the idea of salt stress as a cause for disrupted zones of *Dickinsonia*. Also evidence for land exposure of *Dickinsonia* are recent reports [129, 130] of eolian sedimentary structures: setulfs (obstacle accumulations), wind dissected ripples (transverse scour), climbing translatent stratification (adhesion ripples), and interflag sandstone laminae.

### Trace elements

Analysis of *Dickinsonia* from central and South Australia, and Russian White Sea and Urals show only traces of boron, much lower than in marine rocks. After adjustment for burial alteration and comparison with genuine marine deposits from the same regions, this is evidence that *Dickinsonia* was non-marine [127]. Very early diagenetic cements predating burial compaction of Ediacaran holdfasts in sandstones [131], have Ge/Si ratios >1 μmol/mol characteristic of soil, not aquatic sediment or cements [132]. Dating by ^234^U/^238^U of iron oxides on Ediacaran fossil cover slabs [133] are an inadequate test for recent versus Ediacaran oxidation because the half-life of that rarely used isotopic system, could not reveal Ediacaran age minerals if they were there. There is also evidence for pervasive Ediacaran oxidation of red beds from alternating red and green beds, from claystone breccias with both red and green clasts, from red beds deep in boreholes below green and gray beds, and from tau analysis of ferric and ferrous iron within beds [63, 119, 129, 134].

### Trace fossils

Sequential imprints have been interpreted as trails of motile *Dickinsonia* [2, 135, 136], but are more likely sessile individuals displaced by periglacial frost boils [46, 90], or impressions of “vagrant lichens” or “snow mice” moved intermittently by gusts of wind on ground ice [137–139]. Elongate marks a quarter of the width of the *Dickinsonia* have also been interpreted as trails of movement [45, 140], but that interpretation is precluded by their width disparity. Arcuate marginal lacerations and overfolds are not necessarily evidence of current liftoff [1], but evidence that *Dickinsonia* was attached to the substrate by forces greater than needed to tear the body apart [141]. The nature of *Dickinsonia* attachment to the substrate is revealed by thin sections showing a thick upper pellicle above chambers, but ragged lower boundary with tubular structures down into the matrix [48]. Narrow animal trails consuming *Dickinsonia* were considered scavenging of buried dead bodies [43], but those *Dickinsonia* modules are undecayed and the trails have lateral levees unlike subsurface burrows [142]. *Dickinsonia* shows neither avoidance nor scar-reaction to the attack, which was more likely a case of surface herbivory. Assemblages with *Dickinsonia* and other vendiobionts also show complex rank abundance distribution [143], high β-diversity [144], low interspecific interactions [145], and vegetative propagation [146], unlike modern to Ediacaran or Phanerozoic fossil marine benthic communities [125, 126], and more like terrestrial vegetation [144, 145]. Vendobionts interacted, reproduced, and evolved more like plants and lichens, than like animals.

### Taphonomy

Preservation of *Dickinsonia* and other vendobionts is problematic because they show higher relief than soft-bodied animal fossils, and are preserved more like plants or fungi with burial-compaction-resistant biopolymers such as cellulose, or chitin [3, 10, 42]. The idea of rheological fill beneath a rigid carapace [44] is falsified by lack of internal soft-sediment deformation upwards into the carapace. Instead, thin sections reveal that orthogonal, chambered structure and matrix to filaments below were already partly filled with substrate grains and lacked lamination or other traces of microbial mats [44.48]. Alternatively, relief may have been supported by early diagenetic pyritization [147], or silicification [131].

### Biomarkers

The sponge biomarker 24-isopropylcholesterane is common in indisputably marine Ediacaran rocks of Oman and China, but missing in shales with *Dickinsonia* in Russia [148, 149]. Also in contrast with known Ediacaran marine rocks, Russian shales have (1) unusually high and variable ratio of hopanes/steranes (1.6 to 119, thus variable but generally more bacteria than algae), (2) high and variable δ^15^N (-2.8 ‰ outlier, mostly +3.5 to +6.5 ‰, thus generally without nitrate limitation); (3) high and variable δ^13^Corg (-23.0 to -33.1 ‰, thus cyanobacterial or algal photosynthetic carbon-concentration mechanisms), and (4) low total organic carbon (0.09 to 1.06 wt %, thus highly oxidized). These biomarker levels [from 148, 149] thus support evidence of low boron content [127, 150], that European Vendian shales were deposited in and around lakes or coastal lagoons rather than in the open ocean.

Cholestanes (C27) in *Dickinsonia* [151] are found in animals, but also in fungi and red algae [149]. Cholesterol (C27) is the main sterol in red algae [152, 153]. Glomeromycotan fungi also produce comparable C27 cholesterol [154] and are represented in Ediacaran fossil assemblages by acritarchs [155] and permineralized fragments [156, 157]. Up to 15% cholesterol (C27), along with up to 85% 24-ethyl cholesterol (C29), is present in 5 species of modern symbiotic mycorrhizal *Glomus* (Glomeromycota) [158]. Saprophytic and parasitic fungi with 78–100% cholesterol include *Pneumocysti*s (Ascomycota) [159], *Conidiolobus* (Zygomycota) [160], *Blastocladiella*, *Allomyces* (both Blastocladiomycota] [160], *Rhizophlyctis*, *Monoblepharella* and *Chytridium* (all Chytridiomycota) [161]. This phylogenetic distribution suggests that cholesterol is basal to fungi, and ergosterol (C28) evolved later [160], perhaps before Ediacaran by 650 Ma [153]. Fungal affinities for *Dickinsoni*a may explain the declining ratios of stigmastane/cholestane in progressively larger and older specimens [151–Fig 1D and 1E]. This would not be such a regular pattern if an animal were fouled in old age by green algae with stigmasterol (C29), or if smaller specimens were more affected by local diffusion of algal steroids than larger specimens during burial, but observed regularity is compatible with long-term fungal growth from controlled green algal symbionts with stigmasterol [162]. The balance of steroids, especially lack of C30 steranes in *Dickinsonia* [151], also falsify interpretation as xenophyophore foraminifera [11]. Modern contamination is a concern with the available steroid analyses of Russian *Dickinsonia* [151], considering low amounts of total organic carbon, and weathering of local outcrops, [149]. The virtually unracemized 5β(H) stereochemistry of bacterially-degraded cholesteroid (coprostane), known mainly from animal digestive tracts and sewage [163], is further support for contamination by modern animal feces [149].

## Biological affinities of *Dickinsonia*

Damaged *Dickinsonia* described here rule out animal affinities for *Dickinsoni*a, but not algal or fungal affinities. Ford [164] was first to propose algal affinities for *Charnia*. Other fossils from Charnwood Forest, England, and Mistaken Point, Newfoundland, also have the general appearance and meristematic growth system of algal fronds [26, 55, 86]. Meristematic growth of *Charnia* has been disputed [89], as well as its inclusion with vendobionts [56]. Evidence from steranes of *Dickinsonia* [151] restrict the likely algal group to Rhodophyta [152, 153]. Algal interpretations for vendobionts are unpopular for a variety of reasons: lack of branching bases like algal rhizomorphs, strong relief of the fossils requiring stronger biopolymers than cellulose in algae, lack of mineralization, load-bearing stalks of rangeomorphs tapering upward in a way unable to flex with currents, large internal chambers, within-substrate habit of erniettomorphs, and substrate-hugging habit of dickinsoniamorphs [10, 16, 42, 48].

Similarities of vendobionts with crustose lichens [10] have also are unpopular [2, 151], in part because of different concepts of lichens. A lichen is defined as fungi with symbiotic algae or cyanobacteria, but recent redefinition of lichens as dikaryan fungi only (Ascomycota and Basidiomycota), means that lichens could not be older than Silurian, given palynological lack of evidence for Dikarya before then [165]. Dikaryan lichens have photobionts immobilized by haustorial connections (ectolichens), but lichenized glomeromycotan fungi such as *Geosiphon* engulf the photobionts within a vesicle (endolichens). These differences are comparable with endomycorrhizae and ectomycorrhizae in relationship to their plant hosts [166]. A case has also been made that *Geosiphon* should not be considered a lichen [167], in another attempt to restrict the commonly used term lichen to particular fungal clades and constructions.

**Fig 7:**
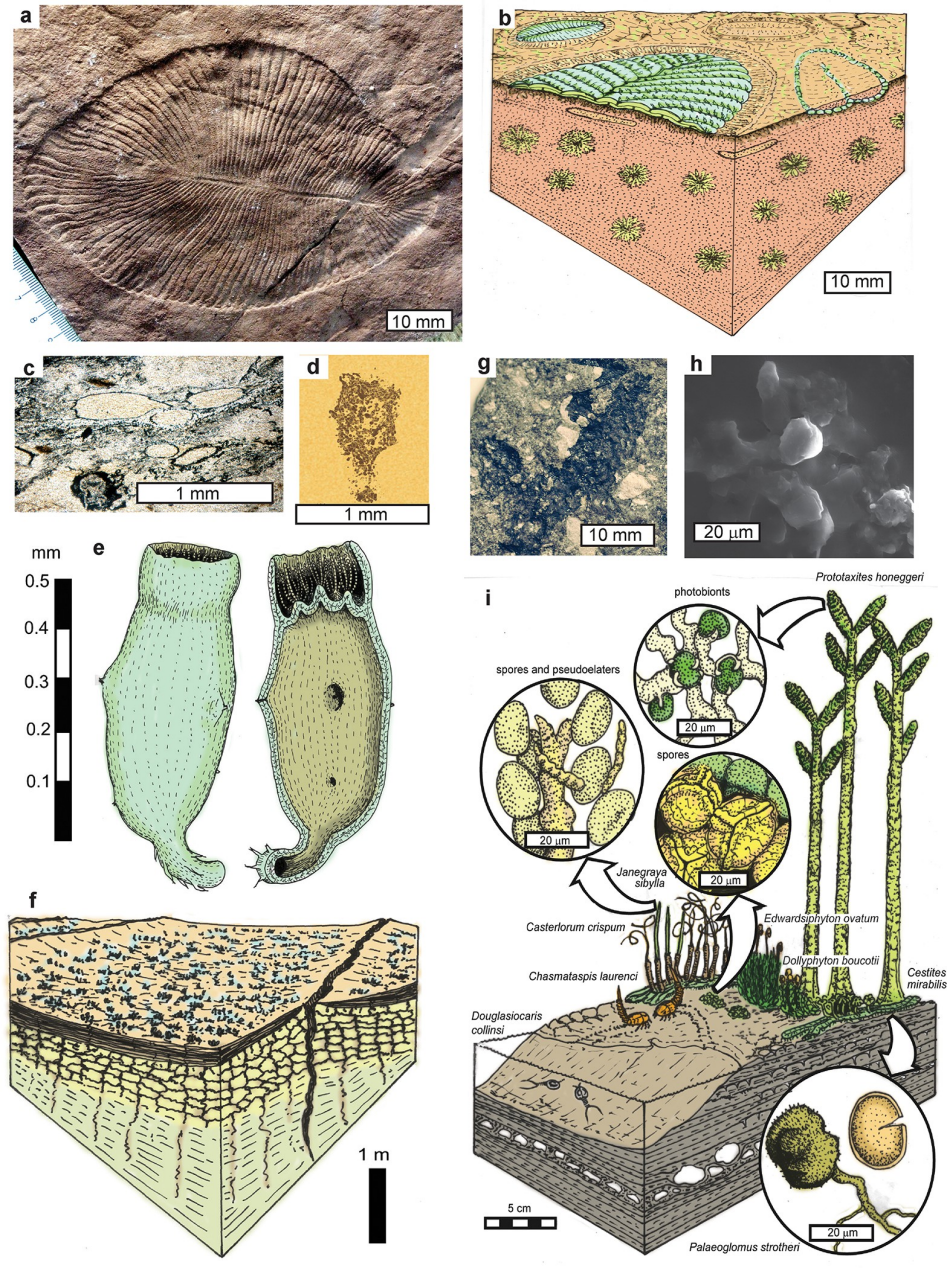
Comparison of *Dickinsonia costata* (a-b), with other extinct lichens, *Diskagma buttonii* from the Palaeoproterozoic (2.1 Ga) upper Hekpoort Basalt near Waterval Onder, South Africa (c-f), and *Prototaxites honeggeri* from the Middle Ordovician (Darriwilian or 460 Ma) Douglas Lake Member, Lenoir Limestone near Douglas Dam, Tennessee (g-i): a, hand specimen; b, reconstruction with *Phyllozoon hanseni* and *Aulozoon* based on thin section study [43, 48]; c, thin section; d, computed x-ray tomography image; e, reconstruction; f, reconstructed paleosol colonized by *Diskagma* [169]; g, branching apex; h, coccoid photobionts gripped by hyphae; i, reconstructed paleosol and associated fossils [85].

*Geosiphon* is a glomeromycotan endolichen with cyanobacterial symbionts enclosed within an interior vesicle [168], similar to 2.1 Ga *Diskagma* (Fig 7C–7F) [169]. Other fossil evidence for glomeromycotan or mucoromycotinan fungi comes from spores as old as 1.5 Ga [155, 170], and permineralized lichens as old as 0.64 Ga [156, 157]. *Geosiphon* and *Diskagma* are plausible glomeromycotan endolichen models for *Dickinsonia* if the internal chambers of *Dickinsonia* (Fig 7A and 7B), housed photosymbionts. Unlike *Geosiphon* however, the photobionts of *Dickinsonia* would have been chlorophyte algae rather than cyanobacteria, judging from sterane biomarkers in *Dickinsonia* [151].

Another plausible models for *Dickinsonia* as an ectolichen are extinct nematophytes, such as *Prototaxites* (Fig 7H and 7I), which had coccoid chlorophyte photobionts with haustorial connections within cortical nests of loose inward-curling hyphae [85, 171]. Similar haustorial connections to coccoid photobionts have also been found in an unnamed early Ediacaran fungus from China [157]. The mainly aseptate hyphae of *Prototaxites* have been interpreted as evidence of glomeromycotan or mucoromycotinan affinities [85, 171]. *Prototaxites* has also been interpreted as an ascomycotan fungus, complete with hymenium [172], which does not appear to be attached to the characteristic nematophyte thallus. Dikaryan affinities are unlikely for small pencil-sized *Prototaxite*s of Ordovician age [85], predating other evidence for Dikarya (Nelsen et al., 2020). By this comparison, *Dickinsonia’s* internal chambers, or “pneu structure” [16], demonstrated in thin section [48], would be comparable with cortical nests of *Prototaxites* [171]. This model for *Dickinsonia* matches the observed relative abundance of green algal stigmasterol and fungal cholesterol in *Dickinsonia* specimens of different inferred individual age [151, 162]. Declining stigmasterol to cholesterol proportions with age are compatible with fungal growth by regulation of green algal photobionts, rather than progressive fouling by algae of an animal, or environmental infiltration. Uncertainty comes from suspected modern contamination of *Dickinsonia* steranes [149, 163].

By either a *Diskagma* or *Prototaxites* model for *Dickinsonia* and other vendobionts, Kingdom Vendobionta [16], demoted to a Class Vendobionta [4], is best placed in fungal divisions Glomeromycota or Mucoromycotina.

## Why was *Dickinsonia* considered marine?

The principal reason why *Dickinsonia* was first considered marine is because Reginald Sprigg, an enthusiastic scuba diver, thought that it looked like sea jelly [173]. This brought him into conflict with his former thesis advisor Sir Douglas Mawson, who also noticed these fossils as enigmatic markings during section measuring [174], but thought that the sandstones were fluvial and associated siltstones were loess [134]. A compromise suggestion of Sprigg, vividly portrayed by Glaessner [175], had them thrown up on the beach by storms. The culmination of this thinking was Peter Trusler’s wonderful reconstruction of *Dickinsonia* as a multicolored worm in shallow oligotrophic tropical waters, an image also featured on Australian postage stamps [98]. Evidence against relationships between *Dickinsonia* and modern marine invertebrates was introduced by Seilacher [16]. Coastal plain and lagoonal habitats were envisaged for *Dickinsonia* by Jenkins et al. [115] and Gehling [116], until the idea that they lived in soils was published [63]. Immediately after that the sedimentary facies of *Dickinsonia* were reinterpreted as entirely subtidal [117, 176], for five reasons: (1) morphological complexity of vendobionts; (2) ripple marks interpreted as marine; (3) massive sandstones interpreted as submarine grain flows; (4) co-occurrence with sea-weed fossils; and (5) similar fossils in China and Australia interpreted as a single marine biotic province. *Dickinsonia* does indeed have regularity of module width and number [42, 45, 93], but lichens and mushrooms also have regularity of form if not damaged (Fig 3D–3F) [10]. Ripple marks form in a variety of marine, lacustrine and fluvial environments, including floodplains [130]. Massive sandstones are not only found in the sea, but deposited by river floods [177, 178]. Algae and other flimsy aquatic plants are fossilized with fossil plants in flood deposits [179, 180]. Plant and lichen remains are also preserved intact within marine and lacustrine deposits [181–183]. China and Australia were closer to each other in the Ediacaran than subsequently [8], at distances allowing shared marine and terrestrial species, judging from Phanerozoic paleogeographic distributions [184].

## Conclusions

Ediacaran *Dickinsonia* specimens from Russia show damage and regeneration that challenges ideas about how they grew, and their biological affinities. A marginal and terminal disrupted zone of wilting forms a necrotic zone separating a regenerated portion, here called an antideltoidal tag, sometimes on two diverging axes rather than a single axis. The nature of the antideltoidal necrotic zone and tag are unlike posterior subterminal regrowth, as in trilobites. The necrotic zone and tag is also unlike regeneration of a posterior tail, as in annelids or millipedes. More likely *Dickinsonia* grew from a deltoid holdfast and elongated by growth from a microscopic antideltoidal apical meristem, which repaired sublethal damage from freezing, salt or sunburn. This meristematic pattern of regrowth found in fungi and plants, is also comparable with growth of other Ediacaran fractal fossils such as *Fractifusus* and *Charnia*. When the apical meristem was damaged within the disrupted zone, lateral meristems formed one or two leaders of antideltoidal tags. The necrotic zone of damage to *Dickinsoni*a is not inflamed, like an infection or frostbite. Nor is it a thick scar or callus, like an amputation. Nor is it a smooth transition to a regenerated limb. The wilted necrotic zone is most like damage by freezing, salt, or sunburn of leaves and lichens, compatible with evidence from associated frigid and gypsic paleosols for life on dusty periglacial soils. *Dickinsonia* grew and regenerated more like fungi and plants, than like animals, and can tentatively be placed within the fungal phyla Mucoromycotina or Glomeromycota.

## References

[1] Evans SD, Droser ML, Gehling JG (2015) *Dickinsonia* liftoff: Evidence of current derived morphologies. Palaeogeography, Palaeoclimatology, Palaeoecology 434: 28–33. 10.1016/j.palaeo.2015.02.006.

[2] Evans SD, Gehling JG, Droser ML (2019) Slime travelers: early evidence of animal mobility and feeding in an organic mat world. Geobiology 17: 490–509. doi: 10.1111/gbi.12351 31180184

[3] Evans SD, Huang W, Gehling JG, Kisailus D, Droser ML (2019) Stretched, mangled, and torn: Responses of the Ediacaran fossil *Dickinsonia* to variable forces. Geology 47: 1049–1053. 10.1130/G46574.1.

[4] Retallack GJ, Broz AP (2020) *Arumberia* and other Ediacaran–Cambrian fossils of central Australia. Historical Biology 32: 1755281. 10.1080/08912963.2020.1755281.

[5] Ivantsov A, Zakrevskaya M, Nagovitsyn A, Krasnova A, Bobrovskiy I, Luzhnaya E (2020) Intravital damage to the body of *Dickinsonia* (Metazoa of the late Ediacaran). Journal of Paleontology 94: 1019–34. 10.1017/jpa.2020.65.

[6] Bobkov NI, Kolesnikov AV, Maslov AV, Grazhdankin DV (2019) The occurrence of *Dickinsonia* in non-marine facies (La aparición de *Dickinsonia* en facies no marinas). Estudios Geologicas 75: e096. 10.3989/egeol.43587.551.

[7] Nesterovsky VA, Martyshyn AI, Chupryna AM (2018) New biocenosis model of Vendian (Ediacaran) sedimentation basin of Podolia (Ukraine). Journal of Geology, Geography, Geoecology 27: 95–107. 10.15421/111835.

[8] Retallack GJ, Matthews N, Master S, Khangar R, Khan M (2021) *Dickinsonia* discovered in India and late Ediacaran biogeography. Gondwana Geology 90: 65–170. 10.1016/j.gr.2020.11.008.

[9] Wang XP, Chen Z, Pang K, Zhou CM, Xiao S, Wan B, et al (2021). *Dickinsonia* from the Ediacaran Dengying Formation in the Yangtze Gorges area, South China. Palaeoworld 30: 602–609 10.1016/j.palwor.2021.01.002.

[10] Retallack GJ (1994). Were the Ediacaran fossils lichens? Paleobiology 20: 523–544. https://www.jstor.org/stable/2401233?seq=1.

[11] Seilacher A, Buatois LA, Mángano MG (2005) Trace fossils in the Ediacaran-Cambrian transition: behavioral diversification, ecological turnover and environmental shift. Palaeogeography, Palaeoclimatology, Palaeoecology 227: 323–356. 10.1016/j.palaeo.2005.06.003.

[12] Valentine J (1992) *Dickinsonia* as a polypoid organism. Paleobiology 18, 378–382. https://www.jstor.org/stable/2400825?seq=1.

[13] Harrington HJ, Moore RC (1956) “Dipleurozoa”, in Treatise on Invertebrate Paleontology. Part F. Coelenterata, Editor Moore R. C. (Boulder and Lawrence, Geological Society of America and University of Kansas Press), F24–26.

[14] Runnegar B (1982) Oxygen requirements, biology and phylogenetic significance of the late Precambrian worm *Dickinsonia*, and the evolution of the burrowing habit. Alcheringa 6: 223–239. 10.1016/j.gr.2020.11.008.

[15] Sperling EA, Vinther J (2010). A placozoan affinity for *Dickinsonia* and the evolution of late Proterozoic metazoan feeding modes. Evolution Development 12:201–209. doi: 10.1111/j.1525-142X.2010.00404.x 20433459

[16] Seilacher A (1992). Vendobionta and Psammocorallia: lost constructions of Precambrian evolution. Journal of the Geological Society of London 149: 607–613. 10.1144/gsjgs.149.4.0607.

[17] Buss LW, Seilacher A (1994) The Phylum Vendobionta: a sister group of the Eumetazoa? Paleobiology 20: 1–4. https://www.jstor.org/stable/2401145?seq=1.

[18] Sugimoto K, Gordon SP, Meyerowitz EM (2011) Regeneration in plants and animals: dedifferentiation, transdifferentiation, or just differentiation? Trends in Cell Biology 21: 212–218. doi: 10.1016/j.tcb.2010.12.004 21236679

[19] Tomlinson PB (1983) Tree architecture: new approaches help to define the elusive biological property of tree form. American Scientist 71 141–149. 17726841

[20] Birnbaum KD, Alvarado AS (2008). Slicing across kingdoms: regeneration in plants and animals. Cell 132: 697–710. doi: 10.1016/j.cell.2008.01.040 18295584 PMC2692308

[21] Honegger R (1995) Experimental studies with foliose macrolichens: fungal responses to spatial disturbance at the organismic level and to spatial problems at the cellular level during drought stress events. Canadian Journal of Botany 73: 569–578. 10.1139/b95-297.

[22] Hammer S (2000). Meristem growth dynamics and branching patterns in the Cladoniaceae. American Journal of Botany 87: 33–47. 10.2307/2656683. 10636828

[23] Sköld M, Rosenberg R (1996) Arm regeneration frequency in eight species of Ophiuroidea (Echinodermata) from European sea areas. Journal of Sea Research 35, 353–362. 10.1016/S1385-1101(96)90762-5.

[24] Godwin JW, Rosenthal N (2014) Scar-free wound healing and regeneration in amphibians: immunological influences on regenerative success. Differentiation 87: 66–75. doi: 10.1016/j.diff.2014.02.002 24565918

[25] Quillen KJ (1998) Ontogenetic scaling of hydrostatic skeletons: geometric, static stress and dynamic stress scaling of the earthworm *Lumbricus terrestris*. Journal of Experimental Biology 201: 1871–1883. doi: 10.1242/jeb.201.12.1871 9600869

[26] Antcliffe JB, Brasier MD (2007). *Charnia* and sea pens are poles apart. Journal of the Geological Society of London 164: 49–51. https://jgs.lyellcollection.org/content/164/1/49.short.

[27] Shen C, Clarkson EN, Yang J, Lan T, Hou JB, Zhang XG (2014) Development and trunk segmentation of early instars of a ptychopariid trilobite from Cambrian Stage 5 of China. Nature Scientific Reports 4: 6970. doi: 10.1038/srep06970 25382488 PMC4225537

[28] Davis RG (1957) Amputations in frostbite. Canadian Medical Association Journal 77: 948–952. 13479849 PMC1824206

[29] Becker MA, Chamberlain JA, Stoffer PW (2000) Pathologic tooth deformities in modern and fossil chondrichthyans: a consequence of feeding‐related injury. Lethaia 33:103–118. 10.1080/00241160050150249.

[30] Bicknell RD, Pates S (2020). Exploring abnormal Cambrian-aged trilobites in the Smithsonian collection. PeerJ 8: e8453. doi: 10.7717/peerj.8453 32117612 PMC7003707

[31] Silberbauer-Gottsberger I, Morawetz W, Gottsberger G (1977). Frost damage of cerrado plants in Botucatu, Brazil, as related to the geographical distribution of the species. Biotropica 9: 253–261. https://www.jstor.org/stable/2388143?seq=1.

[32] Pukacki PM, Przybył K (2005) Frost injury as a possible inciting factor in bud and shoot necroses of Fraxinus excelsior L. Journal of Phytopathology 153: 512–516. 10.1111/j.1439-0434.2005.01010.x.

[33] Inouye DW (2008) Effects of climate change on phenology, frost damage, and floral abundance of montane wildflowers. Ecology 89: 353–362. doi: 10.1890/06-2128.1 18409425

[34] Chang DC, Sohn HB., Cho JH, Im JS, Jin YI, Do GR, et al (2014) Freezing and frost damage of potato plants: a case study on growth recovery, yield response, and quality changes. Potato Research 57: 99–110. 10.1007/s11540-014-9253-5.

[35] Benedict JB (1990) Winter frost injury to lichens: Colorado Front Range. Bryologist 93: 423–426. https://www.jstor.org/stable/3243606?seq=1.

[36] Benedict JB (2009) A review of lichenometric dating and its applications to archaeology. American Antiquity 74: 143–172. https://www.jstor.org/stable/25470542?seq=1.

[37] Leonov MV (2007) Macroscopic plant remains from the base of the Ust’-Pinega formation (Upper Vendian of the Arkhangelsk Region). Paleontological Journal 41: 683–691. 10.1134/S0031030107060123.

[38] Naimark EB, Ivantsov AY (2009) Growth variability in the late Vendian problematics *Parvancorina* Glaessner. Paleontological Journal 43: 12–18. 10.1134/S003103010901002X.

[39] Ivantsov AY, Zakrevskaya MA, Nagovitsyn AL (2019) Morphology of integuments of the Precambrian animals, Proarticulata. Invertebrate Zoology 16: 19–26. 10.15298/invertzool. 16.1.03.

[40] Hoekzema RS, Brasier MD, Dunn FS, Liu AG (2017) Quantitative study of developmental biology confirms *Dickinsonia* as a metazoan. Proceedings of the Royal Society of London B 284: 20171348. doi: 10.1098/rspb.2017.1348 28904140 PMC5597836

[41] Gold DA, Runnegar B, Gehling JG, Jacobs DK (2015) Ancestral state reconstruction of ontogeny supports a bilaterian affinity for *Dickinsonia*. Evolution and Development 17: 315–324. doi: 10.1111/ede.12168 26492825

[42] Retallack GJ (2007) Growth, decay and burial compaction of *Dickinsonia*, an iconic Ediacaran fossil. Alcheringa 31: 215–240. 10.1080/03115510701484705.

[43] Gehling JG, Droser ML (2018) Ediacaran scavenging as a prelude to predation. Emerging Topics in Life Science 2: 213–222. doi: 10.1042/ETLS20170166 32412628

[44] Bobrovskiy I, Krasnova A, Ivantsov A, Luzhnaya (Serezhnikova) E, Brocks JJ (2019) Simple sediment rheology explains the Ediacara biota preservation. Nature Ecology and Evolution 3: 582–589. doi: 10.1042/ETLS20170166 30911145

[45] Ivantsov A, Zakrevskaya M (2021) *Dickinsonia*: mobile and adhered. Geological Magazine, (in press). 10.1017/S0016756821000194.

[46] Retallack GJ (2021) Ediacaran periglacial sedimentary structures. Journal of Palaeosciences 70: 5–30. https://www.bsip.res.in/JPS%20Volume%2070-compressed.pdf.

[47] Wade M (1968) Preservation of soft‐bodied animals in Precambrian sandstones at Ediacara, South Australia. Lethaia 1: 238–267. 10.1111/j.1502-3931.1968.tb01740.x.

[48] Retallack GJ (2016) Ediacaran fossils in thin section. Alcheringa 40: 583–600. 10.1080/03115518.2016.1159412.

[49] Boucot AJ (1990). Evolutionary paleobiology of behavior and coevolution. Elsevier, Amsterdam, 725 p.

[50] Mani MS (2013) Ecology of plant galls. Springer, Berlin, 434 p.

[51] Vick CM, Bevan R (1976) Lichens and tar spot fungus (*Rhytisma acerinum*) as indicators of sulphur dioxide pollution on Merseyside. Environmental Pollution 11: 203–216. 10.1016/0013-9327(76)90085-9.

[52] Tubbs RS, Malefant J, Loukas M., Oakes WJ, Oskouian RJ, Fries FN(2016) Enigmatic human tails: a review of their history, embryology, classification, and clinical manifestations. Clinical Anatomy 29: 430–438. doi: 10.1002/ca.22712 26990112

[53] Janis CM, Bernor RL (2019). The evolution of equid monodactyly: a review including a new hypothesis. Frontiers in Ecology and Evolution 7: e119. 10.3389/fevo.2019.00119.

[54] Müller WA (2002) Autoaggressive, multi-headed and other mutant phenotypes in *Hydractinia echinata* (Cnidaria: Hydrozoa). International Journal of Developmental Biology 46: 1023–1033. www.ijdb.ehu.es. 12533026

[55] Kenchington CG, Dunn FS, Wilby PR (2018) Modularity and overcompensatory growth in Ediacaran rangeomorphs demonstrate early adaptations for coping with environmental pressures. Current Biology 28: 3330–3336. doi: 10.1016/j.cub.2018.08.036 30293718

[56] Dunn FS, Liu AG (2019) Viewing the Ediacaran biota as a failed experiment is unhelpful. Nature Ecology and Evolution 3: 512–514. doi: 10.1038/s41559-019-0815-4 30742104

[57] Niessen FB, Spauwen PH, Schalkwijk J, Kon M (1999) On the nature of hypertrophic scars and keloids: a review. Plastic Reconstructive Surgery 104: 1435–1458. doi: 10.1097/00006534-199910000-00031 10513931

[58] Hoffmann F, Rapp HT, Zöller T, Reitner J (2003) Growth and regeneration in cultivated fragments of the boreal deep water sponge *Geodia barretti* Bowerbank, 1858 (Geodiidae, Tetractinellida, Demospongiae). Journal of Biotechnology 100: 109–118. doi: 10.1016/s0168-1656(02)00258-4 12423905

[59] Lavrov AI, Bolshakov FV, Tokina DB, Ereskovsky AV (2018) Sewing up the wounds: The epithelial morphogenesis as a central mechanism of calcaronean sponge regeneration. Journal of Experimental Zoology Part B: Molecular Development and Evolution 330: 351–371. doi: 10.1002/jez.b.22830 30421540

[60] Srivastava M, Begovic E, Chapman J, Putnam NH, Hellsten U, Kawashima T, et al (2008). The *Trichoplax* genome and the nature of placozoans. Nature 454: 955–960. doi: 10.1038/nature07191 18719581

[61] Ramon-Mateu J, Ellison ST, Angelini TE, Martindale MQ (2019) Regeneration in the ctenophore Mnemiopsis leidyi occurs in the absence of a blastema, requires cell division, and is temporally separable from wound healing. BMC Biology 17, 1–25. doi: 10.1186/s12915-019-0695-8 31604443 PMC6788111

[62] Bely AE, Nyberg KG (2010) Evolution of animal regeneration: re-emergence of a field. Trends in Ecology and Evolution 25: 161–170. doi: 10.1016/j.tree.2009.08.005 19800144

[63] Retallack GJ (2013) Ediacaran life on land. Nature 493: 89–92. doi: 10.1038/nature11777 23235827

[64] Heidtmann B, Brandt O (2003) Burns and sunburn. In Abeck D, Burgdorf W., editors, Common Skin Diseases in Children. Steinkopff, Heidelberg, Steinkopff, pp. 31–38. 10.1007/978-3-7985-1966-4_5.

[65] Oglesby LC (1969) Salinity-stress and desiccation in intertidal worms. American Zoologist 9: 319–331. 10.1093/icb/9.2.319.

[66] Briggs DE (1995) Experimental taphonomy. Palaios 10: 539–550. 10.2307/3515093.

[67] Racskó J, Szabó T, Nyéki J, Soltész M, Nagy PT (2010) Characterization of sunburn damage to apple fruits and leaves. International Journal of Horticultural Science 16: 15–20. 10.31421/IJHS/16/4/909.

[68] Shapira OR, Israeli Y, Shani URI, Schwartz A (2013) Salt stress aggravates boron toxicity symptoms in banana leaves by impairing guttation. Plant Cell Environment 36: 275–287. doi: 10.1111/j.1365-3040.2012.02572.x 22765264

[69] Thiemann M, Ruthmann A (1991) Alternative modes of asexual reproduction in *Trichoplax adhaerens* (Placozoa). Zoomorphology 110, 165–174. 10.1007/BF01632872.

[70] Ereskovsky AV, Tokina DB (2007) Asexual reproduction in homoscleromorph sponges (Porifera; Homoscleromorpha). Marine Biology 151:425–434. 10.1007/s00227-006-0439-5.

[71] Diaz JA, Movilla J, Ferriol P (2019) Individualistic patterns in the budding morphology of the Mediterranean demosponge *Aplysina aerophoba*. Mediterranean Marine Science 20: 282–286. 10.12681/mms.19322.

[72] Fischer AB, Hofmann DK (2004) Budding, bud morphogenesis, and regeneration in *Carybdea marsupialis* Linnaeus, 1758 (Cnidaria: Cubozoa). Hydrobiologia 530: 331–337. 10.1007/s10750-004-2658-4.

[73] Bałuk W, Radwański A (1984) New data on the Korytnica Basin, its organic communities and ecological relationships between species (Middle Miocene; Holy Cross Mountains, Central Poland). Acta Geologica Polonica 34: 179–194.

[74] Iten HV, Cox RS (1992) Evidence of clonal budding in a radial cluster of *Paraconularia crustula* (White) (Pennsylvanian:? Cnidaria). Lethaia 25: 421–426. 10.1111/j.1502-3931.1992.tb01645.x.

[75] Barr JI, Somaweera R, Godfrey SS, Gardner MG, Bateman PW (2020) When one tail isn’t enough: abnormal caudal regeneration in lepidosaurs and its potential ecological impacts. Biological Reviews 95: 1479–1496. doi: 10.1111/brv.12625 32583608

[76] Lindsay SM (2010) Frequency of injury and the ecology of regeneration in marine benthic invertebrates. Integrative and Comparative Biology 50: 479–493. doi: 10.1093/icb/icq099 21558216

[77] Minelli A, Fusco G (2004) Evo-devo perspectives on segmentation: model organisms, and beyond. Trends in Ecology and Evolution 19: 423–429. doi: 10.1016/j.tree.2004.06.007 16701300

[78] Alibardi L 2019 The regenerating tail blastema of lizards as a model to study organ regeneration and tumor growth regulation in amniotes. The Anatomical Record 302: 1469–1490. doi: 10.1002/ar.24029 30421533

[79] Fusco G (2005) Trunk segment numbers and sequential segmentation in myriapods. Evolution and Development 7: 608–617. doi: 10.1111/j.1525-142X.2005.05064.x 16336414

[80] Honegger R (1993) Developmental biology of lichens. New Phytologist 125: 659–677. doi: 10.1111/j.1469-8137.1993.tb03916.x 33874446

[81] Seminara A, Fritz J, Brenner MP, Pringle A (2018) A universal growth limit for circular lichens. Journal Royal Society London Interface 15, 20180063. doi: 10.1098/rsif.2018.0063 29875282 PMC6030627

[82] Sanders WB, de Los Ríos A (2017). Parenchymatous cell division characterizes the fungal cortex of some common foliose lichens. American Journal of Botany 104: 207–217. doi: 10.3732/ajb.1600403 28202453

[83] Ferrini F (2006) Pollarding and its effects on tree physiology: a look to mature and senescent tree management in Italy. Colloque Européen sur les Trognes 26: 1–8.

[84] Lewis CE (1906) The embryology and development of *Riccia lutescens* and *Riccia crystallina*. Botanical Gazette 41: 109–138.

[85] Retallack GJ (2019) Ordovician land plants and fungi from Douglas Dam, Tennessee. Palaeobotanist 68: 173–205. https://blogs.uoregon.edu/gregr/detailed-webpage/publications/.

[86] Laflamme M, Narbonne GM, Greentree C, Anderson MM (2007) Morphology and taphonomy of an Ediacaran frond: Charnia from the Avalon Peninsula of Newfoundland. In Vickers-Rich P, Komarower P, editors, The Rise and Fall of the Ediacaran Biota. Geological Society of London Special Publication 286, pp. 237–257.

[87] Liu AG, McIlroy D, Matthews JJ, Brasier MD (2013) Exploring an Ediacaran ‘nursery’: growth, ecology and evolution in a rangeomorph palaeocommunity. Geology Today 29: 23–26. 10.1111/j.1365-2451.2013.00860.x.

[88] Stewart HL (2008) The role of spatial and ontogenetic morphological variation in the expansion of the geographic range of the tropical brown alga, *Turbinaria ornata*. Integrative and Comparative Biology 48: 713–719. doi: 10.1093/icb/icn028 21669827

[89] Dunn FS, Liu AG, Donoghue PCJ (2018) Ediacaran developmental biology. Biological Reviews 93: 914–921. doi: 10.1111/brv.12379 29105292 PMC5947158

[90] Retallack GJ (2016) Field and laboratory tests for recognition of Ediacaran paleosols. Gondwana Research 36: 107–123. 10.1016/j.gr.2016.05.001.

[91] Gehling JG, Droser ML, Jensen SR, Runnegar BN (2005) Ediacara organisms: relating form to function. In Briggs DEG. Editors, Evolving form and function: fossils and development: proceedings of a symposium honouring Adolf Seilacher for his contributions to paleontology. Yale University Press, New Haven, 43–67.

[92] Brasier MD, Antcliffe JB (2008) *Dickinsonia* from Ediacara: a new look at morphology and body construction. Palaeogeography, Palaeoclimatology, Palaeoecology, 270: 311–323. 10.1016/j.palaeo.2008.07.018.

[93] Evans SD, Droser ML, Gehling JG (2017) Highly regulated growth and development of the Ediacara macrofossil *Dickinsonia costata*. PLoS One 12, e0176874. doi: 10.1371/journal.pone.0176874 28520741 PMC5435172

[94] Glaessner MF, Wade M (1971). *Praecambridium* ‐ a primitive arthropod. Lethaia 4: 71–77. 10.1111/j.1502-3931.1971.tb01280.x.

[95] Zakrevskaya MA, Ivantsov AY (2017) *Dickinsonia costata*–the first evidence of neoteny in Ediacaran organisms. Invertebrate Zoology 14: 92–98. 10.15298/invertzool.14.1.13.

[96] Wade M (1972) *Dickinsonia*: polychaete worms from the late Precambrian Ediacara fauna, South Australia. Queensland Museum Memoir 16: 171–190.

[97] Jacobs DK, Hughes NC, Fitz-Gibbon ST, Winchell CJ (2005) Terminal addition, the Cambrian radiation and the Phanerozoic evolution of bilaterian form. Evolution and Development 7: 498–514. doi: 10.1111/j.1525-142X.2005.05055.x 16336405

[98] Fedonkin MA, Gehling JG, Grey K, Narbonne GM, Vickers-Rich P (2007) The rise of animals: evolution and diversification of the kingdom Animalia. Johns Hopkins University Press, Baltimore.

[99] Schierwater B, Kuhn K (1998) Homology of Hox genes and the zootype concept in early metazoan evolution. Molecular Phylogenetics and Evolution 9: 375–381. doi: 10.1006/mpev.1998.0489 9667985

[100] Eitel M, Guidi L, Hadrys H, Balsamo M, Schierwater B (2011) New insights into placozoan sexual reproduction and development. PloS One 6, e19639. doi: 10.1371/journal.pone.0019639 21625556 PMC3098260

[101] Feuda R, Dohrmann M, Pett W, Philippe H, Rota-Stabelli O, Lartillot N, et al (2017) Improved modeling of compositional heterogeneity supports sponges as sister to all other animals. Current Biology 27:3864–3870. doi: 10.1016/j.cub.2017.11.008 29199080

[102] Zhao Y, Vinther J, Parry LA, Wei F, Green E, Pisani D, et al (2019) Cambrian sessile, suspension feeding stem-group ctenophores and evolution of the comb jelly body plan. Current Biology 29: 1112–1125. doi: 10.1016/j.cub.2019.02.036 30905603

[103] Cavalier-Smith T (2004) Only six kingdoms of life. Proceedings of the Royal Society of London B 271: 1251–1262. doi: 10.1098/rspb.2004.2705 15306349 PMC1691724

[104] Torruella G, De Mendoza A, Grau-Bové X, Antó M, Chaplin MA, Del Campo J, et al (2015) Phylogenomics reveals convergent evolution of lifestyles in close relatives of animals and fungi. Current Biology 25: 2404–2410. doi: 10.1016/j.cub.2015.07.053 26365255

[105] Burki F, Roger AJ, Brown MW, Simpson AG (2020). The new tree of eukaryotes. Trends in Ecology and Evolution 35: 43–55. doi: 10.1016/j.tree.2019.08.008 31606140

[106] Hake S, Smith HM, Holtan H, Magnani E, Mele G, Ramirez J (2004) The role of knox genes in plant development. Annual Revies of Cell and Developmental Biology 20: 125–151. doi: 10.1146/annurev.cellbio.20.031803.093824 15473837

[107] Duboule D (2007) The rise and fall of Hox gene clusters. Development 134:, 2549–2560. doi: 10.1242/dev.001065 17553908

[108] Thangavel G, Nayar S (2018) A survey of MIKC type MADS-box genes in non-seed plants: algae, bryophytes, lycophytes and ferns. Frontiers in Plant Science 9: 00510. doi: 10.3389/fpls.2018.00510 29720991 PMC5915566

[109] Drost HG, Janitza P, Grosse I, Quint M (2017) Cross-kingdom comparison of the developmental hourglass. Current Opinion on Genetics and Development 45: 69–75. doi: 10.1016/j.gde.2017.03.003 28347942

[110] Mumby HS, Chapman SN, Crawley JA, Mar KU, Htut W, Soe AT, et al (2015) Distinguishing between determinate and indeterminate growth in a long-lived mammal. BMC Evolutionary Biology 15: 1–9. doi: 10.1186/s12862-015-0487-x 26464339 PMC4604763

[111] Wilkinson PM, Rainwater TR, Woodward AR, Leone EH, Carter C (2016) Determinate growth and reproductive lifespan in the American alligator (*Alligator mississippiensis*): evidence from long-term recaptures. Copeia 104: 843–852. 10.1643/CH-16-430.

[112] Omeyer LC, Fuller WJ, Godley BJ, Snape RT, Broderick AC (2018) Determinate or indeterminate growth? Revisiting the growth strategy of sea turtles. Marine Ecology Progress Series 596:199–211. 10.3354/meps12570.

[113] Hariharan IK, Wake DB, Wake MH (2016) Indeterminate growth: could it represent the ancestral condition?. Cold Spring Harbor Perspectives in Biology 8: a019174. 10.1101/cshperspect.a019174. PMC4743077 26216720

[114] Gehling JG, Narbonne GM (2007) Spindle-shaped Ediacara fossils from the Mistaken Point assemblage, Avalon zone, Newfoundland. Canadian Journal of Earth Sciences 44: 367–387. 10.1139/e07-003.

[115] Jenkins RJF, Ford CH, Gehling JG (1983) The Ediacara Member of the Rawnsley Quartzite: the context of the Ediacara assemblage (late Precambrian, Flinders Ranges). Journal of the Geological Society of Australia 30: 101–119. 10.1080/00167618308729240.

[116] Gehling JG (2000) Environmental interpretation and a sequence stratigraphic framework for the terminal Proterozoic Ediacara Member within the Rawnsley Quartzite, South Australia. Precambrian Research 100: 65–95. 10.1016/S0301-9268(99)00069-8.

[117] Gehling JG, Droser ML (2013) How well do fossil assemblages of the Ediacara Biota tell time? Geology 41: 447–450. 10.1130/G33881.1.

[118] Grazhdankin D (2004) Patterns of distribution in the Ediacaran biotas: facies versus biogeography and evolution. Paleobiology 30: 203–221. 10.1666/0094-8373(2004)030<0203:PODITE>2.0.CO;2.

[119] Retallack GJ (2012) Were Ediacaran siliciclastics of South Australia coastal or deep marine? Sedimentology 59: 1208–1236. 10.1111/j.1365-3091.2011.01302.x.

[120] Retallack GJ, Broz AP (2020) Late Ediacaran and Cambrian paleosols from central Australia. Palaeogeography Palaeoclimatology Palaeoecology 560: 110047. 10.1016/j.palaeo.2020.110047.

[121] Retallack GJ (2011) Problematic megafossils in Cambrian paleosols of South Australia. Palaeontology 54: 1223–1242. 10.1111/j.1475-4983.2011.01099.x.

[122] Retallack GJ (2016c) Ediacaran sedimentology and paleoecology of Newfoundland reconsidered. Sedimentary Geology 333: 15–31. 10.1016/j.sedgeo.2015.12.001.

[123] Liu AG, Dunn FS (2020). Filamentous connections between Ediacaran fronds. Current Biology 30: 1322–1328. doi: 10.1016/j.cub.2020.01.052 32142705

[124] Tarhan LG, Droser M L, Gehling JG, Dzaugis MP (2017) Microbial mat sandwiches and other anactualistic sedimentary features of the Ediacara Member (Rawnsley Quartzite, South Australia): implications for interpretation of the Ediacaran sedimentary record. Palaios 32: 181–194. 10.2110/palo.2016.060.

[125] Smith EF, Nelson LL, Strange MA, Eyster AE, Rowland SM, Schrag DP, et al (2016) The end of the Ediacaran: Two new exceptionally preserved body fossil assemblages from Mount Dunfee, Nevada, USA. Geology 44:.911–914. 10.1130/G38157.1.

[126] Schiffbauer JD, Huntley JW, O’Neil GR, Darroch SA, Laflamme M, Cai Y (2016) The latest Ediacaran wormworld fauna: Setting the ecological stage for the Cambrian explosion. GSA Today 26(11): 4–11. 10.1130/GSATG265A.1.

[127] Retallack GJ (2020) Boron paleosalinity proxy for deeply buried Paleozoic and Ediacaran fossils. Palaeogeography, Palaeoclimatology, Palaeoecology 540: 109536. 10.1016/j.palaeo.2019.109536.

[128] Retallack GJ (2021) Towards a glacial subdivision of the Ediacaran Period, with example of the Boston Bay Group, Massachusetts. Australian Journal of Earth Sciences 10.1080/08120099.2021.1954088.

[129] McMahon WJ., Liu AG, Tindal BH, Kleinhans MG(2020) Ediacaran life close to land: coastal and shoreface habitats of the Ediacaran macrobiota, the central Flinders Ranges, South Australia. Journal of Sedimentary Research 90: 1463–1499. 10.2110/jsr.2020.029.

[130] Retallack GJ (2019) Interflag sandstone laminae, a novel fluvial sedimentary structure with implication for Ediacaran paleoenvironments. Sedimentary Geology 379: 60–76. 10.1016/j.sedgeo.2018.11.003.

[131] Tarhan LG, Hood AV, Droser ML, Gehling JG, Briggs D E (2016) Exceptional preservation of soft-bodied Ediacara Biota promoted by silica-rich oceans. Geology 44: 951–954. 10.1130/G38542.1.

[132] Retallack GJ (2017) Exceptional preservation of soft-bodied Ediacara Biota promoted by silica-rich oceans: comment. Geology 44: e407. 10.1130/G38763C.1.

[133] Tarhan LG, Planavsky NJ, Wang X, Bellefroid EJ, Droser ML, Gehling JG (2018) The late‐stage “ferruginization” of the Ediacara Member (Rawnsley Quartzite, South Australia): Insights from uranium isotopes. Geobiology 16: 35–48. doi: 10.1111/gbi.12262 29105940

[134] Mawson D, Segnit ER (1949) Purple slates of the Adelaide System. Transactions of the Royal Society of South Australia 72: 276–280.

[135] Ivantsov AY (2011) Feeding traces of proarticulata—the Vendian metazoa. Paleontological Journal 45: 237–248. 10.1134/S0031030111030063.

[136] Ivantsov AY (2013) Trace fossils of Precambrian metazoans “Vendobionta” and “Mollusks”. Stratigraphy and Geological Correlation 21: 252–264. 10.1134/S0869593813030039.

[137] Pérez FL (1994) Vagrant cryptogams in a paramo of the high Venezuelan Andes. Flora 189: 263–276. 10.1016/S0367-2530(17)30601-1.

[138] Pérez FL (2020) Andean rolling mosses gather on stone pavements: Geoecology of *Grimmia longirostris* Hook. in a high periglacial páramo. Catena 187:104389. 10.1016/j.catena.2019.104389.

[139] Hotaling S, Bartholomaus TC, Gilbert SL (2020) Rolling stones gather moss: Movement and longevity of moss balls on an Alaskan glacier. Polar Biology 43: 735–744. 10.1007/s00300-020-02675-6.

[140] Ivantsov A, Nagovitsyn A, Zakrevskaya M (2021) Traces of locomotion of Ediacaran macroorganisms. Geosciences 9: e395. 10.3390/geosciences9090395.

[141] Retallack GJ (2017) Comment on: “*Dickinsonia* liftoff: evidence of current derived morphologies” by Evans S. D., Droser M. L., and Gehling J.G. Palaeogeography, Palaeoclimatology, Palaeoecology 485: 999–1001. 10.1016/j.palaeo.2015.07.005.

[142] Buatois LA, Mángano MG (2016) Ediacaran ecosystems and the dawn of animals. In Mángano MG, Buatois LA, editors,The trace-fossil record of major evolutionary events. Springer, Dordrecht, pp. 27–72. 10.1007/978-94-017-9600-2_2.

[143] Darroch SA, Laflamme M, Wagner PJ (2018) High ecological complexity in benthic Ediacaran communities. Nature Ecology and Evolution 2: 1541. doi: 10.1038/s41559-018-0663-7 30224815

[144] Finnegan S, Gehling JG, Droser ML (2019) Unusually variable paleocommunity composition in the oldest metazoan fossil assemblages. Paleobiology 45: 1–11. 10.1017/pab.2019.1.

[145] Mitchell EG, Butterfield NJ (2018) Spatial analyses of Ediacaran communities at Mistaken Point. Paleobiology 44: 40–57. doi: 10.1017/pab.2017.35

[146] Mitchell EG, Kenchington CG, Liu AG, Matthews JJ, Butterfield NJ (2015) Reconstructing the reproductive mode of an Ediacaran macro-organism. Nature 524: 343–346. doi: 10.1038/nature14646 26237408

[147] Liu AG, McMahon S, Matthews JJ, Still JW, Brasier AT (2019) Petrological evidence supports the death mask model for the preservation of Ediacaran soft-bodied organisms in South Australia. Geology 47: 215–218. 10.1130/G45918.1.

[148] Pehr K, Love GD, Kuznetsov A, Podkovyrov V, Junium CK, Shumlyanskiy L, et al (2018) Ediacara biota flourished in oligotrophic and bacterially dominated marine environments across Baltica. Nature Communications 9: e1807. doi: 10.1038/s41467-018-04195-8 29728614 PMC5935690

[149] Love GD, Zumberge JA (2021) Emerging patterns in proterozoic lipid biomarker records. Cambridge University Press, Cambridge 10.1017/9781108847117.

[150] Pirrus EA (1992) Freshening of the late Vendian basin on the East European Craton. Estonian Academy of Sciences Geology Proceedings 41: 115–123.

[151] Bobrovskiy I, Hope JM, Ivantsov A, Nettersheim BJ, Hallmann C, Brocks JJ (2018) Ancient steroids establish the Ediacaran fossil *Dickinsonia* as one of the earliest animals. Science 361: 1246–1249. doi: 10.1126/science.aat7228 30237355

[152] Fattorusso E, Magno S, Santacroce C, Sica D, Impellizzeri G, Mangiafico S, et al (1975) Sterols of some red algae. Phytochemistry 14: 1579–1582. 10.1016/0031-9422(75)85354-4.

[153] Gold DA (2018) The slow rise of complex life as revealed through biomarker genetics. Emerging Topics in Life Science 2: 191–199. doi: 10.1042/ETLS20170150 32412622

[154] Weete JD, Abril M, Blackwell M (2010) Phylogenetic distribution of fungal sterols. PloS One 5(5): e10899. doi: 10.1371/journal.pone.0010899 20526375 PMC2878339

[155] Retallack GJ (2015) Acritarch evidence of a late Precambrian adaptive radiation of Fungi. Botanica Pacifica 4: 19–33. 10.17581/bp.2015.0420.

[156] Yuan X, Xiao S, Taylor TN (2005) Lichen-like symbiosis 600 million years ago. Science 308: 1017–1020. doi: 10.1126/science.1111347 15890881

[157] Gan T, Luo T, Pang K, Zhou C, Zhou G, Wan B, et al (2021) Cryptic terrestrial fungus-like fossils of the early Ediacaran Period. Nature Communications 12: 641. doi: 10.1038/s41467-021-20975-1 33510166 PMC7843733

[158] Grandmougin-Ferjani A, Dalpé Y, Hartmann MA, Laruelle F, Sancholle M (1999) Sterol distribution in arbuscular mycorrhizal fungi. Phytochemistry 50: 1027–1031. 10.1016/S0031-9422(98)00636-0.

[159] Kaneshiro ES, Wyder MA (2000) C27 to C32 sterols found in Pneumocystis, an opportunistic pathogen of immunocompromised mammals. Lipids 35: 317–324. doi: 10.1007/s11745-000-0528-8 10783009

[160] Weete JD, Gandhi SR (1997) Sterols of the phylum Zygomycota: phylogenetic implications. Lipids 32: 1309–1316. doi: 10.1007/s11745-006-0169-y 9438242

[161] Weete JD, Fuller MS, Huang MQ, Gandhi S (1989). Fatty acids and sterols of selected hyphochytriomycetes and chytridiomycetes. Experimental Mycology 13: 183–195. 10.1016/0147-5975(89)90023-6.

[162] Kodner RB, Pearson A, Summons RE, Knoll AH (2008) Sterols in red and green algae: quantification, phylogeny, and relevance for the interpretation of geologic steranes. Geobiology 6:.411–420. doi: 10.1111/j.1472-4669.2008.00167.x 18624688

[163] Summons RE, Erwin DH (2018) Chemical clues to the earliest animal fossils. Science 361:1198−1199. doi: 10.1126/science.aau9710 30237342

[164] Ford TD (1958) Pre-Cambrian fossils from Charnwood Forest. Proceedings of the Yorkshire Geological Society 31: 211–217. 10.1144/pygs.31.3.211.

[165] Nelsen MP, Lücking R, Boyce CK, Lumbsch HT, Ree RH (2020) No support for the emergence of lichens prior to the evolution of vascular plants. Geobiology 18: 3–13. doi: 10.1111/gbi.12369 31729136

[166] Chilvers GA, Lapeyrie FF, Horan DP (1987) Ectomycorrhizal vs endomycorrhizal fungi within the same root system. New Phytologist 107: 441–448. doi: 10.1111/j.1469-8137.1987.tb00195.x 33873840

[167] Hawksworth DL (1988) The variety of fungal-algal symbioses, their evolutionary significance and the nature of lichens. Botanical Journal of the Linnaean Society London 96: 3–20. 10.1111/j.1095-8339.1988.tb00623.x.

[168] Schüßler A. (2002) Molecular phylogeny, taxonomy, and evolution of Geosiphon pyriformis and arbuscular mycorrhizal fungi. In Smith SE, Smith FA, editors, Diversity and Integration in Mycorrhizas. Springer, Dordrecht, pp. 75–83. 10.1007/978-94-017-1284-2_8.

[169] Retallack GJ, Krull ES, Thackray GD, Parkinson D (2013) Problematic urn-shaped fossils from a Paleoproterozoic (2.2 Ga) paleosol in South Africa. Precambrian Research 235: 71–87. 10.1016/j.precamres.2013.05.015.

[170] Loron CC, François C, Rainbird RH, Turner EC, Borensztajn S, Javaux EJ (2019) Early fungi from the Proterozoic era in Arctic Canada. Nature 570: 232–235. doi: 10.1038/s41586-019-1217-0 31118507

[171] Retallack GJ, Landing E (2014) Affinities and architecture of Devonian trunks of Prototaxites loganii. Mycologia 106: 1143–1158. doi: 10.3852/13-390 24990121

[172] Honegger R, Edwards D, Axe L, Strullu-Derrien C (2018) Fertile *Prototaxites taiti*: a basal ascomycete with inoperculate, polysporous asci lacking croziers. Philosophical Transactions of the Royal Society of London B 373: e20170146. doi: 10.1098/rstb.2017.0146 29254969 PMC5745340

[173] Sprigg R (1989) Geology is Fun: Recollections. Arkaroola, Adelaide, 349 p.

[174] Mawson D (1938) Cambrian and sub-Cambrian formations at Parachilna Gorge. Transactions of the Royal Society of South Australia 62: 255–262.

[175] Glaessner MF (1961) Pre-Cambrian animals. Scientific American 204: 72–78. https://www.jstor.org/stable/24937392?seq=1.13738093

[176] Xiao S, Droser M, Gehling JG, Hughes IV, Wan B, Chen Z, et al (2013) Affirming life aquatic for the Ediacara biota in China and Australia. Geology 41: 1095–1098. 10.1130/G34691.1.

[177] Conaghan PJ, Jones JG (1975) The Hawkesbury Sandstone and the Brahmaputra: a depositional model for continental sheet sandstones. Journal of the Geological Society of Australia 22: 275–283. 10.1080/00167617508728897.

[178] Jones BG, Rust BR (1983) Massive sandstone facies in the Hawkesbury Sandstone, a Triassic fluvial deposit near Sydney, Australia. Journal of Sedimentary Research 53: 1249–1259. 10.1306/212F8355-2B24-11D7-8648000102C1865D.

[179] Krings M, Klavins SD, Barthel M, Lausberg S, Serbet R, Taylor TN, et al (2007) *Perissothallus*, a new genus for Late Pennsylvanian-Early Permian noncalcareous algae conventionally assigned to *Schizopteris* (aphleboid foliage). Botanical Journal of the Linnean Society London 153: 477–488. 10.1111/j.1095-8339.2007.00616.x.

[180] Retallack GJ, Dilcher DL (2012) Outcrop versus core and geophysical log interpretation of mid-Cretaceous paleosols from the Dakota Formation of Kansas. Palaeogeography, Palaeoclimatology, Palaeoecolology 329: 47–63. 10.1016/j.palaeo.2012.02.017.

[181] Retallack GJ (1985) Triassic fossil plant fragments from shallow marine rocks of the Murihiku Supergroup, New Zealand. Journal of the Royal Society of New Zealand 15: 1–26. 10.1080/03036758.1985.10421741.

[182] Taylor TN, Krings M, Taylor EL 2015 Fossil Fungi. Academic Press (Elsevier), London, San Diego, Waltham, and Oxford, p. 382.

[183] Friedman M, Carnevale G (2018) The Bolca Lagerstätten: shallow marine life in the Eocene. Journal of the Geological Society of London 175: 569–579. 10.1144/jgs2017-164.

[184] Smith P {1988] Paleoscene# 11. Paleobiogeography and plate tectonics. Geoscience Canada 15: 261–279. https://id.erudit.org/iderudit/geocan15_4art0.

